# Diffusion of treatment in social networks and mass drug administration

**DOI:** 10.1038/s41467-017-01499-z

**Published:** 2017-12-05

**Authors:** Goylette F. Chami, Andreas A. Kontoleon, Erwin Bulte, Alan Fenwick, Narcis B. Kabatereine, Edridah M. Tukahebwa, David W. Dunne

**Affiliations:** 1 Department of Land Economy, University of Cambridge, 19 Silver St., Cambridge, CB3 9EP UK; 2 Department of Pathology, University of Cambridge, Tennis Ct. Rd., Cambridge, CB2 1QP UK; 3 Development Economics Group, Wageningen University, Hollandseweg 1, Wageningen, 6706 KN The Netherlands; 4 Schistosomiasis Control Initiative, Imperial College London, Norfolk Pl., London, W2 1PG UK; 5 Uganda Ministry of Health, Vector Control Division, Bilharzia and Worm Control Programme, 15 Bombo Rd., Kampala, Uganda

## Abstract

Information, behaviors, and technologies spread when people interact. Understanding these interactions is critical for achieving the greatest diffusion of public interventions. Yet, little is known about the performance of starting points (seed nodes) for diffusion. We track routine mass drug administration—the large-scale distribution of deworming drugs—in Uganda. We observe friendship networks, socioeconomic factors, and treatment delivery outcomes for 16,357 individuals in 3491 households of 17 rural villages. Each village has two community medicine distributors (CMDs), who are the seed nodes and responsible for administering treatments. Here, we show that CMDs with tightly knit (clustered) friendship connections achieve the greatest reach and speed of treatment coverage. Importantly, we demonstrate that clustering predicts diffusion through social networks when spreading relies on contact with seed nodes while centrality is unrelated to diffusion. Clustering should be considered when selecting seed nodes for large-scale treatment campaigns.

## Abstract (web summary)

Mass drug administration depends on the distributors’ contact with community members. Using data of deworming treatment distribution from Ugandan villages, the authors show that community medicine distributors with tightly-knit friendship connections achieve the greatest reach and speed of coverage.

## Introduction

The social connections between people influence the spread of information, behaviors, and technologies^1–5^. Understanding the structure of social networks is essential for targeting beneficiaries and improving the diffusion of public interventions^6^. A key policy challenge concerns how best to identify and to evaluate the performance of starting points (seed nodes) for interventions. Seed nodes in social networks are predominantly selected and targeted based on their centrality^7–9^. Centrality is a set of measurements that describes the connectivity and embeddedness of a node in a network and is widely viewed as a measure of power and influence in social systems^10^. High centrality of seed nodes promotes simple epidemic spreading, which is the most common model of diffusion^5,11^. In these models, each additional social contact increases the probability of information transmission and diffusion unfolds along the shortest paths in a network^12,13^. Long, weak links that bridge otherwise disconnected groups facilitate diffusion^14,15^, but this simple epidemic spreading^5,11^ does not account for possible brokerage properties of bridges in social networks^16^. On the other hand, the reach of seed nodes has been shown to rely on the topology of the network^3,4,17,18^. Influential seed nodes may group together^3^ or belong to the core of the network^17^. Network topology is particularly important in models of complex contagion^19^, where repeated exposure and social reinforcement are needed before an individual partakes in an intervention. In contrast to simple epidemic models, the probability of information or technologies spreading is zero until an exposure threshold is met for the recipient^12,13^ and diffusion tends to travel along short densely clustered ties^1,2,4,19,20,21,22^. Clustering is one indicator of network transitivity and is pervasive in social networks^23^. Transitivity implies that two nodes are connected if they share a network neighbor^24^. In friendship networks, clustering exists if two friends of the node of interest also are friends. Importantly, in simple epidemic models, high network transitivity causes redundancy and slows spreading, whereas in complex contagion models, this redundancy is required for diffusion to occur.

It is not known whether centrality (simple epidemic) or transitivity (complex contagion) is relevant for evaluating seed node performance in real-world contexts that require repeated contact with seed nodes. Diffusion that relies on repeated contact with seed nodes applies to a number of important social interventions in rural poor areas of low-income countries. For example, the distribution of bed nets, vaccines, and deworming treatments in low-income countries depends on a small set of locally trained individuals and their repeated contact with other community members^25,26^. These seed nodes are tasked with approaching all households at specified time points to administer medicines as opposed to relying on a chance probability of meeting. Thus far, the study of seed nodes in social networks has assumed that after the initial subset of individuals are informed by the seed nodes, there is no need for subsequent individuals to directly contact seed nodes. An important open question remains: what determines spreading when seed nodes must directly contact potential recipients? Herein, we address this question by examining the diffusion of treatment through mass drug administration (MDA) and village friendship networks.

MDA is the community-wide distribution of preventive chemotherapies to treat human parasitic worms^26^. Treatments are provided at no financial cost and are safely administered to infected and uninfected individuals. Annual or biannual MDA is necessary due to rapid reinfection. In sub-Saharan Africa, over 633 million people require preventive chemotherapy, yet only 1/3rd of these individuals are approached for treatment through MDA^27^. Each village in our study area in Mayuge District, Uganda has two community medicine distributors (CMDs). These individuals are unpaid volunteers^28^, who receive only a nominal reimbursement for transport to training. CMDs are trained and retrained annually to treat all eligible individuals in their village. In 2013, an undisturbed, routine round of MDA was tracked for 1 month. This time frame enabled comparisons across study villages, minimized recall bias for treatment coverage outcomes, and was within national treatment schedules. Data were collected in 17 villages for 16,357 individuals within 3491 homes. Friendship networks were defined at the household level because CMDs were instructed by the local government to move from home to home. An undirected edge between two households was generated if a member of one household named any member of another household as a close friend. The advantages of studying close friendship networks for MDA include the lack of predefined roles and network positions of CMDs, as well as an approximation of static networks in our rural villages. A static network has edges that do not change frequently (in this case, close friendships remained over several years).

Our study design avoids several potential sources of bias and provides a real-world environment to examine seed node performance. MDA for intestinal schistosomiasis and soil-transmitted helminths began in our study area in 2003^26^. Accordingly, the target population was familiar with MDA, social learning was unnecessary, and most individuals would comply with treatment if offered in this context^29^. Before the first round of MDA in our study area, government health workers visited communities and asked villagers to meet and select CMDs^30^. This context is the real setting that public interventions must be assessed. Selecting any person, e.g., via random assignment, to be a seed node often is not possible. To integrate stakeholders of interventions, government or non-governmental organizations utilize individuals selected by their communities^7,26^. Researchers and village members knew the starting points for information and treatment diffusion. The current CMDs had been active for several years and were reminded of their duties during annual retraining. CMDs were well acquainted with their responsibilities and identifiable by other villagers. Apart from CMDs, eligible individuals had no access to MDA pills or information about drug availability outside of their village. Nationally led information campaigns and school-based distribution had not commenced during the study. Hence, information on drug availability was initially monopolized by CMDs. Per routine MDA procedures, CMDs received training and drugs for MDA outside of their village. Treatments were only available from CMDs within the village. Nearby health centers and primary schools had not received drugs during the study (due to delays by the national control program) and CMDs did not leave pills with treated households to pass onwards to untreated households. Also, CMDs had knowledge of and registered all households prior to the start of distribution and were provided a sufficient number of pills to treat all individuals in their village.

The treatment coverage achieved by CMDs was examined. In each village, we measured coverage as the percentage of eligible households that were visited and offered treatment by CMDs. Specifically, household coverage (binary) was defined as at least one eligible person in the home being approached by CMDs and offered at least one treatment through MDA. Positive household coverage included eligible individuals who were visited by CMDs and refused treatment. Treatment coverage measured the delivery of medicines. Coverage was studied to isolate CMD performance (as opposed to the ingestion of pills; compliance) simply because the choice to offer medicine to eligible individuals rests with the CMD, whereas compliance with the offered treatment is dependent on the final decision of the recipient. To identify what factors determined the total reach and speed of household coverage in each village, we assessed measures of centrality^10^ and local transitivity^31^ of seed nodes. These network indicators were normalized to account for variation in village/network size and then averaged for the two CMDs in each village. For the speed of diffusion, the number of days required for CMDs to reach 50% household coverage was examined. This 50% target is a highly conservative measure of World Health Organization (WHO) guidelines, which recommend at least 75% coverage of all school-aged children as a feasible goal^25^. The 50% target was chosen to include as many villages as possible while remaining as close as possible to actual WHO goals. We find that clustering predicts the reach and speed of drug delivery, while centrality is unrelated to diffusion. Clustering should be considered when selecting seed nodes for large-scale treatment campaigns.

## Results

### Network completeness and external consistency

Complete, sociocentric networks were constructed that included a total of 3436 nodes and 16,155 edges (Supplementary Table 1). In all villages, if the household did not belong to the main connected component, the household was an isolate (belonged to no other network components). Across the 17 villages, only 55 households were not in the main component and 87 households refused to participate in the survey. Therefore, 96.03% (3436/3578) of all households available at the time of survey were included in the main component of their village network. Only 4.95% (859/17347) of names provided in all villages for friendship connections—the maximum possible edges, including self-loops and multi-edges—were not matched; a remarkable 99.04% (16200/16357) of individuals who were recorded in household surveys were in a household included in a main network component. No upward bias, which would entail individuals naming as many outgoing connections as possible irrespective of friendship status, was observed. Only 4.83% (166/3436) of households included in the friendship networks listed the maximum of 10 outgoing connections.

**Fig. 1:**
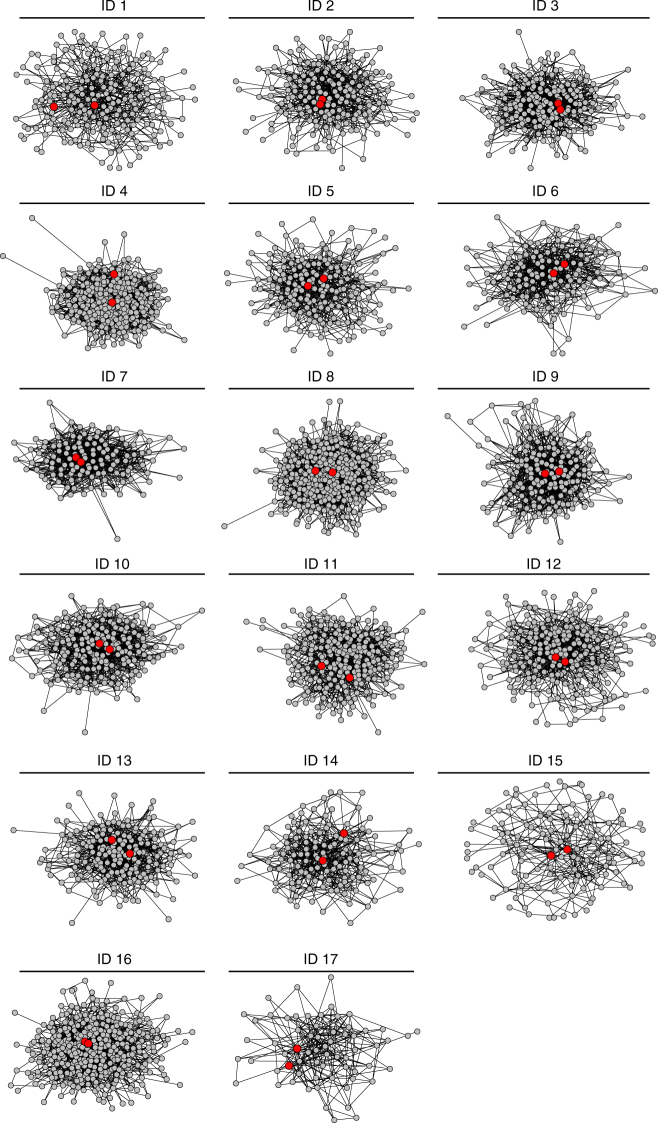
Community medicine distributors in full village friendship networks. Figure 1 presents friendship networks for each of the 17 study villages. The numbers above each graph are the village IDs. The red nodes represent the location of the community medicine distributors (CMDs) in the network. In all villages, CMDs are well connected and embedded in the center of their village friendship network. Full descriptions, including the number of nodes and edges, are provided in Supplementary Table 1

**Fig. 2:**
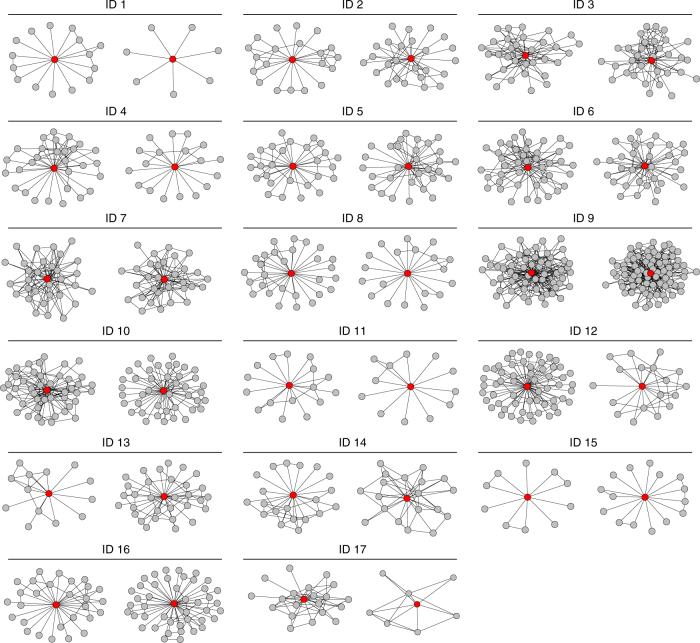
Egocentric networks of community medicine distributors. Figure 2 presents 34 egocentric networks where the ego of interest is the community medicine distributor (CMD). There were two CMDs per village and the numbers above the graphs correspond to the village ID. These plots are not ordered by any descriptive or network characteristics. The subgraphs were extracted from full village networks and each neighbor of the CMD and all corresponding edges between those neighbors were included. Vertices in these networks are households and edges indicate friendship ties between households. The CMD household is the red vertex in the center of the subgraph. Wide variation in clustering—the frequency that friends of CMDs also were friends—is easily observable across villages. For example, the star and star-like ego networks in village ID 1 have clustering coefficients of 0 and 0.067 and the ego networks of village ID 7 have clustering coefficients of 0.168 and 0.191

The networks studied here share structural properties with a wide range of complex physical, biological, and social systems (Supplementary Table 1 and Supplementary Fig. 1)^32^. High centrality was observed for CMDs (Fig. 1), who were selected by members of their village. The degree of each CMD varied considerably and ranged from 7 to 80. However, CMD degree was greater, in every village, than the average degree of all households. As shown in Fig. 2, wide variation was observed in the local transitivity of connections among CMDs and their friends. The clustering coefficient of CMDs varied greatly from star-shaped ego networks (clustering coeff. = 0; village ID 1) to highly transitive ego networks (clustering coeff. = 0.429; village ID 17). See Supplementary Table 2 for a summary of CMD network statistics.

### Treatment coverage achieved

In the study villages, household coverage varied from 23.14 to 93.53% (Supplementary Tables 3, 4). Overall, approximately one-third (1011/3415) of households were not approached by CMDs during 1 month of MDA. There was little or no informational diffusion about drug availability between households that were visited by CMDs and households that were never approached for treatment. When non-recipients were asked if they heard that drugs were available during the 1-month distribution, 85.06% (860/1011) of households that were not offered medicines by CMDs had no eligible person in the home that knew drugs were available in the village. These households constituted 25.18% (860/3415) of all households with at least one person who was eligible for treatment through MDA.

Clustering, due to its positive correlation with the number of households visited by CMDs, was associated with increased treatment coverage. The frequency that friends of CMDs were also friends (clustering) was positively related to household coverage (*p* value = 0.011) (Table 1). CMDs in the 90th percentile of clustering were estimated to have approached 9.32% (*p* value < 0.001) more households in their village, achieving on average 78.1% household coverage, when compared to CMDs in the 10th percentile of clustering who reached an estimated 68.8% of households. Hence, clustering was associated with a village realizing the 75% coverage target set by the WHO^25^. This target is an approximation of the treatment coverage needed to control morbidity attributable to helminthic infections. Our results are consistent with findings from artificially constructed online networks. Centola shows how global (network-level) clustering is efficient for the spread of social behaviors^1^. Global (village level) clustering was associated with average CMD clustering (Supplementary Fig. 2) and household coverage (Supplementary Table 5). Though, when global and CMD clustering were compared in stepwise regressions, using an alpha-to-enter of 0.15, only average CMD clustering was retained as a significant predictor of household coverage.

**Table 1:** Influence of CMD network properties on village treatment coverage

Although CMDs with high clustering achieved the greatest household coverage, the number or completeness of connections among their friends was not informative. The proportion of possible edges (density) was borderline insignificant (*p* value = 0.055) and the fraction of reciprocated ties among friends of CMDs was uncorrelated (*p* value >0.05) with household coverage. Belonging to a well-connected group of friends was not associated with household coverage; the largest degree shared among the friends of CMDs (core number) was insignificant (*p* value >0.05). There was no support (*p* value >0.05) that CMDs needed to belong to a large set of households, where everyone was completely connected to everyone else in the friend group, i.e., have a high clique number.

Social network analysis in low-income countries has relied on centrality for guiding the selection of seed nodes^8^. For example, in a project in India, seed nodes were selected non-randomly by a non-governmental organization and used to spread information about an innovation—microfinance^7^. The communication and eigenvector centralities of these first-informed seed nodes were positively associated with the reach of diffusion, although which seed nodes actually shared information was unknown. We found no such relationship in our study. CMD centrality indicators, which measured neighborhood size, connectivity, embeddedness, and accessibility to other nodes, were unrelated (*p* value >0.05) to household coverage. Our results suggest that being well connected or embedded in a network may facilitate the diffusion of innovations with simple epidemic spreading^7^, but was not informative for the diffusion of a familiar technology, where spreading relied on contact with seed nodes (our study). There is experimental support for this inference from Honduras, where well-connected nodes did not achieve greater diffusion of multivitamins when compared to randomly selected nodes^33^. We also find no correlation of clustering with centrality (Supplementary Table 6). Clustering as opposed to centrality should be considered when selecting seed nodes for existing technologies and diffusion processes that depend on repeated contact with seed nodes.

### Speed of treatment distribution

**Fig. 3:**
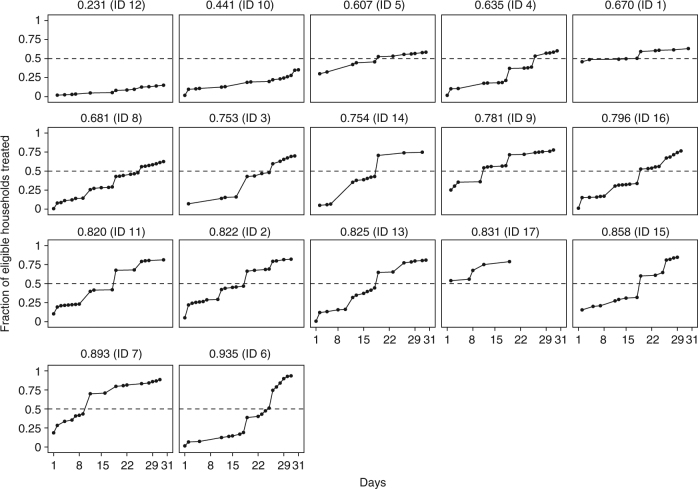
Time series of household treatment coverage. In Fig. 3, household coverage is the cumulative fraction of eligible households where at least one eligible individual was offered at least one drug through mass drug administration. Information on the day of drug receipt was unavailable for individuals refusing treatment. These noncompliers were excluded from the calculation of treatment delivery speed (see Supplementary Methods). To enable a comparison with the reach of household coverage, the plots are ordered by household coverage achieved, which is stated in the graph titles. Household coverage may differ from the graph maximum. Household coverage included noncompliers and individuals who were offered and accepted treatment but did not remember the day of drug receipt. In parentheses, the village ID is presented. The dashed line represents 50% household treatment. Village ID 17, which had a shorter distribution span than the other villages, was the smallest village of only 65 households. This village also approached 50% of households in the first day of distribution. As a robustness check, the speed analysis was repeated for individual coverage, which was the proportion of eligible individuals offered treatment (Supplementary Fig. 3 and Supplementary Table 8). In this case, village ID 17 approached 50% of eligible individuals on Day 7

The speed of treatment distribution was assessed by comparing the number of days required for each village to achieve 50% household coverage (Fig. 3). Two of five villages (ID 7 and ID 17) that achieved the highest household coverage were also among the five fastest villages. The frequencies of households offered treatment against the day of CMD visits are provided in Supplementary Table 7. Three indicators of local transitivity were associated with the speed of treatment distribution—clustering, reciprocity, and density (Table 2). CMDs with tightly knit friends (high clustering) were more likely (*p* value = 0.015) to approach 50% of households sooner than CMDs with friends who were not connected (low clustering). The fraction of reciprocated edges in the CMD ego networks was associated (*p* value = 0.045) with faster diffusion of household coverage. CMDs with higher edge density in their ego networks were more likely (*p* value = 0.013) to achieve 50% household coverage in fewer days than CMDs whose friends were less densely connected. These relationships imply that increasing local transitivity for CMDs may halve the distribution time to reach 50% household coverage in a 1-month period of MDA. The predicted time to reach 50% household coverage was 10.48–11.93 days shorter (*p* value <0.001) for CMDs at the 90th percentiles of average clustering, reciprocity, or density when compared to CMDs at the 10th percentiles of these network properties. These results suggest that seed nodes are most efficient, with respect to diffusion speed, when located within clusters^34^. This practical finding can help policymakers conceptualize the time needed to reach targets when diffusion is reliant on clustering. We find that the delivery of medicines occurs much slower than recommended by WHO guidelines, which suggests that treatment coverage of 75% of individuals is a feasible goal in routine schedules set by local governments for MDA^25^. In our study region in Uganda, these routine schedules aim to complete drug distribution within 2–3 weeks (Supplementary Methods). Yet, during 1 month of MDA, only 64.71% (11/17) and 25% (3/17) of villages had CMDs who approached, respectively, 75% of households or 75% of eligible individuals for at least one treatment.

The core and clique numbers of CMDs as well as several indicators of CMD centrality were uncorrelated (*p* > 0.05) with the number of days required to reach 50% household coverage. All results remained when reanalyzed against the proportion of eligible individuals offered treatment (Supplementary Table 8 and Supplementary Fig. 3).

### Robustness to confounders

We studied possible confounding factors (Supplementary Table 9). Temporal clustering, which was measured as the standard deviation of the day of drug receipt for households connected to CMDs, was insignificant (*p* value >0.05) for household coverage. Thus, the influence of clustered friends on CMD performance cannot be attributed to CMDs quickly and directly communicating with all of their friends about ongoing MDA^35^. Differences in diffusion among villages were not simply due to the lack of communication between CMDs. The CMDs, who achieved the greatest diffusion, did not need to be close friends. An edge (direct connection) between seed nodes was insignificantly (*p* value >0.05) associated with household coverage. The maximum shortest path length (geodesic distance) connecting two CMDs was three edges and variation in this small social distance was uncorrelated (*p* value >0.05) with household coverage. Incentives to conform^36^ or to receive financial payment were highly unlikely as CMDs were unpaid volunteers who were trained to promote MDA. No apparent difference in the abilities of CMDs to distribute treatment was observed. All CMDs were better off with respect to socioeconomic status and network position than the rest of their village (Supplementary Tables 3, 4) and the capacity of CMDs, as measured by degree or closeness centrality, was uncorrelated with diffusion (Tables 1, 2). Hence, community selection successfully identifies well-connected and better-educated people to be CMDs, but can be improved by also incorporating clustering into the selection criteria.

**Table 2:** Influence of CMD network properties on speed of treatment diffusion

We also explored whether the presence of homophily—the shared likeness among CMDs and their friends—confounded and inflated effects of CMD network properties^37,38^. We found no evidence (*p* value >0.05) that homophily affected the reach of household coverage (Supplementary Table 9). All results of temporal clustering, CMD friendship, and homophily remained robust against village treatment distribution speed (Supplementary Table 10). Moreover, the shared likeness and personal characteristics of CMDs in each village also did not affect our results or CMD clustering, respectively (Supplementary Tables 11–13).

**Fig. 4:**
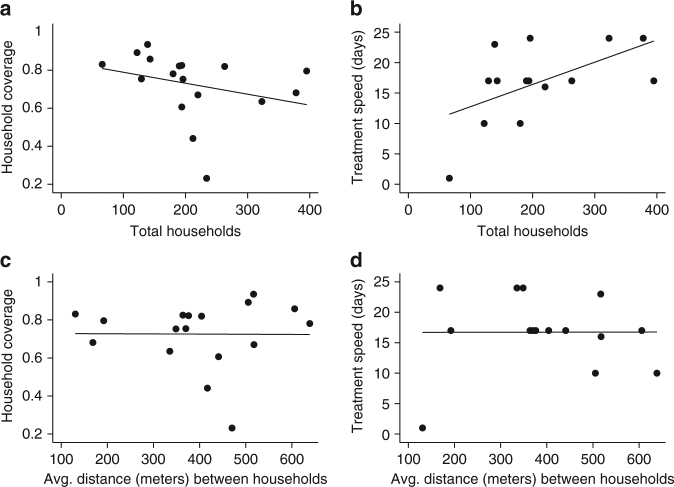
Village size and accessibility against treatment outcomes. Household coverage is defined as the proportion of households in the village where at least one eligible person in the home was offered at least one treatment through mass drug administration. Treatment speed is defined as the number of days until community medicine distributors visited 50% of households. Within the one-month distribution period, 15/17 villages achieved 50% household coverage. Supplementary Table 14 presents the regression results and significance of the relationships shown in Fig. 4. **a**, **b** The village size, as measured by the total households in the village, was not significantly related to household coverage (*p* value = 0.077) and borderline insignificant for treatment speed (*p* value = 0.054). **c**, **d** The average distance between any two households in each village was calculated in meters. There was no correlation (*p* value >0.05) with household coverage or treatment speed and the average distance between households

Lastly, Fig. 4 and Supplementary Table 14 present the association of village size, accessibility, and ecology with treatment coverage and speed. The total number of households was unrelated (*p* value >0.05) to the household coverage achieved and was borderline insignificant (*p* value = 0.054) for how quickly 50% of households were approached. Moreover, the proportion of total households directly connected to CMDs in the village friendship network was uncorrelated (*p* value >0.05) with household coverage and treatment delivery speed. Villages with few households (e.g., IDs 9, 15) were not necessarily small with respect to geographical spread (Supplementary Fig. 4). The physical size of the village, which was measured as the furthest distance in meters between two households (spatial diameter), was unrelated (*p* value >0.05) to diffusion. Concerning village accessibility, no correlation (*p* value >0.05) of household coverage or treatment delivery speed with the average distance in meters between two households or village ecology was observed. In addition to moving from home to home to distribute treatments, as instructed by the national program during training, 41.18% (7/17) of villages had CMDs who also informed individuals to come to their home to pick up treatments. This increased availability or access to treatments was uncorrelated (*p* value > 0.05) with diffusion.

## Discussion

Seeding effective distribution of treatment by CMDs relies on clustering. Strong ties among friends of CMDs were associated with an increased rate and level that households were visited and offered treatment by CMDs. Unlike simple epidemic processes that require only one contact to propagate information, seed nodes required constant reinforcement to engage in effective drug distribution. Social reinforcement to deliver treatment is needed for CMDs, who are volunteers, due to the costly investment of time required to visit and revisit all households^39^. Ex-post focus groups with CMDs and village leaders revealed one mechanism of clustering that increased drug delivery by CMDs (Supplementary Methods). Close-knit friends were better able to provide information to the CMDs about problems in the community and missed households. We isolated the mechanism of clustering from other roles^35,40^ of social reinforcement such as ostracism of poor-performing CMDs, an explanation that was dismissed by focus group participants.

Clustered friends of CMDs were better able to provide social reinforcement as the closure in the network structure facilitated direct communication between the friends^40^. These findings are consistent with models of complex contagion^1,19,20^. Our clustering result also extends and develops published empirical findings^41,42^, which show that local networks around women determine contraceptive use in low-income countries, to the diffusion of medicines targeting all individuals in a village. Additional studies in other countries are recommended to externally validate these findings and to further analyze causality.

Future treatment plans should consider clustering when selecting seed nodes and designing interventions to improve diffusion. Graphing complete sociocentric village networks, as we did in this study, is an expensive and arduous task for MDA programs in low-income countries. However, our observation that clustering is associated with the diffusion of treatment suggests that the whole village need not be graphed. Targeting clustering is relatively simple, as only the direct connections are needed for CMDs or other seed nodes (ego networks). To further simplify measuring clustering in routine assessments by MDA program managers, future research could investigate the accuracy and applicability of proxy indicators for clustering. Such proxy indicators may include CMDs’ perceived ego networks and nominations of individuals with high clustering by village members.

Clustering can guide public interventions for MDA. MDA programs, in particular for onchocerciasis^43–45^, have demonstrated success by utilizing kinship structures in rural communities. The selection of CMDs by kinship has reduced CMD attrition, increased treatment coverage, and held CMDs accountable for providing health information. Additional work should investigate the extent in which such social structures may overlap with friendship networks. Friendship networks are another social structure that can complement kinship and be used to improve CMD selection. During the initial selection of seed nodes, village-nominated CMDs can be surveyed to assess if their friends are connected. The final selection of CMDs can be informed by how tightly knit the connections are among their friends. In villages with established CMDs, interventions to improve treatment coverage can focus on increasing clustering. Joint social activities or shared tasks can be established for friends of CMDs to foster connections within this group. These friends may also be asked to serve as a feedback loop of information to CMDs about community perceptions of ongoing problems with drug delivery. This potential role of friends in monitoring the village may partially alleviate opportunity costs that are incurred by volunteer CMDs from time spent distributing medicines.^28^. Alternatively, additional seed nodes with high clustering can be selected to assist existing CMDs with treatment distribution and to disseminate information to CMDs and the wider community.

## Methods

### Ethics

This study was reviewed and approved by the Uganda National Council of Science and Technology, the Office of the President in Uganda, and the Cambridge University Human Biological Research Ethics Committee. Informed consent was obtained from all respondents and recorded on tablet computers preceding the household interview. All village names have been replaced with village codes to preclude the identification of individuals in the study villages.

### Participants and data source

On 1 November 2013, household surveys commenced in the 17 study villages following 1 month of undisturbed MDA. CMDs were not informed of these visits beforehand to avoid influencing distribution and the surprise visits were explained as an effort to understand the efficacy of the national drug programs on infection prevalence. Households in each village were approached for interview and information was collected on every person in the home that was at least 1 year of age. The household head was interviewed and, if married, a spouse also was asked to participate. Socioeconomic information and treatment receipt outcomes were surveyed for every household member, and network data were collected at the household level. Data were collected on a total of 16,357 individuals in 3491 households. Infants were not included in the household list. Preliminary work, in February 2013, to understand naming conventions (how children are named when born, how female names change when they marry, and so on) showed that adults did not count individuals younger than 1 year as a household member. Moreover, individuals younger than 1 year were ineligible for any of the drugs that were distributed through MDA, per WHO guidelines^46^.

### Treatment coverage outcomes

Treatment coverage was measured for all 16,357 participants by asking if praziquantel, albendazole, or ivermectin was offered to them from CMDs within the past month. Three methods were used to facilitate the recognition of the drugs. Photos (Supplementary Fig. 5) of each drug were shown on tablet computers, detailed descriptions were provided, and the actual drugs were handed to respondents to touch, see, and smell. If an individual stated they received a drug, then a follow-up question asking which day in the past month the individual received the drugs was asked (Supplementary Table 15). To assist individuals with understanding time, familiar events such as a school week, day of church attendance, and so on were informally provided as examples of time reference points. Directly observed treatment (DOT)^47^ is the standard procedure for all neglected tropical diseases targeted by MDA in Uganda. In DOT, CMDs are trained to assist and to observe an individual swallowing the offered medicines. CMDs are retrained on DOT each year during training conducted by district health personnel. Though quantitative evidence is unavailable to confirm the enforcement and monitoring of DOT, self-reported compliance (drug ingestion) suggests high conformity to DOT. Over 88% (7358/8302) of 16,200 individuals in friendship networks (see Supplementary Methods), who were offered medicine, swallowed the drugs offered. Anecdotally, CMDs indicated that such ingestion was observed, i.e., DOT was used. However, the Ugandan national control program does not equate the distribution of medicines to actual ingestion, a frequent assumption of global MDA statistics. This inference is a result of anecdotal reports outside of our study period of individuals convincing CMDs to deviate from DOT practices and to allow them to swallow the medicines at another time.

Varying definitions of treatment coverage complicate comparisons of studies across the MDA literature^47–50^. Our definition here is most similar to the WHO recommended definition of surveyed coverage. However, the difference between treatment coverage in this paper and the definition of surveyed coverage^47,48^ is that treatment coverage here included individuals who refused to ingest pills. Our indicator captured an important aspect of CMD performance, which is not the targeted measurement of WHO definitions that focus on recipient outcomes. We directly measured how often CMDs make available and offer medicines to eligible individuals. This outcome would be underestimated if we relied on surveyed coverage. For comparison, we recalculated household coverage as surveyed coverage, i.e., measured household coverage as positive only when at least one eligible individual in the home self-reported ingestion of drugs. This surveyed coverage was nearly perfectly correlated with our outcome (Pearson’s *r* = 0.967, *p* value < 0.001). Thus, our results can be compared to studies using surveyed coverage.

### Network construction

The nodes and edges in the network, respectively, were households and friendship ties. An edge was generated between households if any person in a household was named or had named any person in another household as a close friend. A self-loop occurred when a household named someone within their own home as a close friend. Households were instructed to name individuals outside of their home. Accordingly, self-loops were ignored and only households belonging to the main component were analyzed. Multiple connections (multi-edges) in the same direction from one household to another household, e.g., naming multiple people from the same household, were measured as one edge. We make the working assumption that villages are independent; though it is possible some individuals may have friends in other villages in the study. The full set of 16,357 individuals in the 17 villages was used in the network construction. The name generator was the following:

Please tell me the clan name first then the second name of up to 10 people that are very close friends to you. You should feel comfortable to turn to this person to borrow tools for fishing or farming without paying. A close friend is also someone that you see frequently. Do not name anyone in your household. Provide the names in the order of who is your closest friend first. Only name people in your village.

### Statistical analysis

**Fig. 5:**
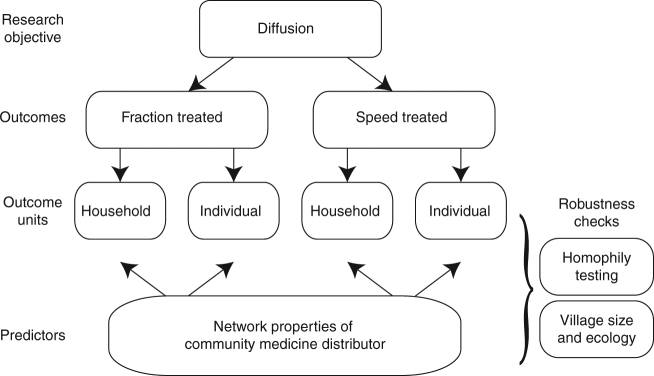
Schematic of the statistical analysis. The main outcome of interest was diffusion. This outcome was measured as the maximum fraction of eligible households or individuals offered treatment by community medicine distributors (CMDs) and the speed at which those households or individuals were offered treatment. Speed measured which villages reached a specific level of treatment coverage and at which day in the distribution period. The network characteristics of CMDs were the main predictors of interest. To understand why certain network indicators of CMDs may be associated with the diffusion outcomes, robustness checks were conducted. The contribution of homophily to diffusion was assessed, i.e., the affinity for treatment or shared factors that may predict treatment coverage and are unrelated to network topology. The robustness checks also included examining village ecology, size, and physical spread of households (in meters) against diffusion

Treatment coverage, which represented the performance of CMDs, was analyzed using Stata v.13.1. Figure 5 presents the diffusion outcomes for CMDs and an overview of the statistical analysis. Diffusion achieved by CMDs was measured at the village level by examining the fraction of the village that was offered treatment by CMDs and the speed at which treatment was distributed. These variables, as defined in the treatment coverage section of the Supplementary Methods, were constructed for both households and individuals to capture different aspects of diffusion and to test the consistency of the results. Household coverage, which measured if at least one eligible individual in the home was offered treatment by CMDs, indicated if the CMD approached the home. Household coverage was the focus of this paper. As a robustness check, individual coverage also was examined. Individual coverage represented the proportion of eligible individuals offered treatment by CMDs in a village. This variable may capture if a CMD returned to a home to provide treatment to individuals who were not present on the first visit or to administer the second round of treatment (albendazole and ivermectin). Topological characteristics of centrality and clustering for the CMD households in the full village friendship network were used to explain diffusion in robust^51^ fractional response models with Probit estimation^52^ and Poisson regressions^53^. Additional robustness checks are provided in the Supplementary Methods and Supplementary Tables 16–21.

### Data availability

All relevant data are available in the paper, Supplementary Information, and on request from the corresponding authors.

## Electronic supplementary material

Supplementary Information
