# Supplementary material for: Diffusion of treatment in social networks and mass drug administration
Source: Nat Commun. 2017 Dec 5;8:1929. doi: 10.1038/s41467-017-01499-z (PMC5717046; doi:10.1038/s41467-017-01499-z)
Supplement: Supplementary file 1 — Supplementary Information [file 41467_2017_1499_MOESM1_ESM.pdf]

Supplementary Methods

Study area

This study was conducted in Mayuge District, Uganda from September to November 2013 in the context of a routine, annual round of mass drug administration (MDA). During this time period, MDA was implemented in all districts. Seventeen villages were surveyed within five kilometers of Lake Victoria. This catchment was chosen as it was guaranteed praziquantel treatment by the national control programme due to known transmission of intestinal schistosomiasis (*Schistosoma mansoni*). Concerning intestinal helminths, the study area was only endemic with *S. mansoni* and hookworm infections, although the hookworm species is unknown [1,2]. Praziquantel and albendazole distribution commenced in Mayuge District in 2003 and 2006, respectively, and was repeated annually. The selected parishes (one administrative unit below a district and above a village) received praziquantel and albendazole for at least two consecutive years preceding the study. The history of ivermectin treatment was not available because researchers and local health officials did not locate any written records, as of February 2013, in the district health office of Mayuge.

To ensure comparability of villages based on wealth and infrastructural development (Supplementary Table 16), the study villages were chosen as follows. In February 2013, a preliminary survey of 41 villages in Mayuge District was conducted. This survey identified where villages were located within the five-kilometer catchment that was eligible for praziquantel treatment. Waypoints were recorded at the centre of each village. To assess infrastructure, the village chairman identified the location of public latrines, taps, swamps, primary schools, religious facilities, and health centres. This information was used to select 17 villages that were similar in infrastructure, size, home materials, number of neighbouring villages, distance to government health facilities, and distance to large towns with established markets. As per the local language, all study villages shared a common language of Lusoga and had the same majority tribes of Mudama or Musoga.

Timeline of mass drug administration

In the last two weeks of September 2013, the district Vector Control Officer (VCO) trained community medicine distributors (CMDs) in accordance with routine procedures for mass drug administration (MDA). The VCO met with the CMDs outside of their villages in nearby government health centres or primary schools. The CMDs were taught or retrained to register, to determine eligibility, and to administer correct treatment dosages [3]. The VCO provided new national treatment registers and sufficient quantities of praziquantel, albendazole, and ivermectin pills to treat everyone in the CMDs' villages. At this time, the VCO confirmed CMDs had knowledge of all households in their villages as the CMDs had previously registered all homes for MDA. CMDs were instructed to complete distribution of praziquantel in the first week of October and to complete administration of a package of albendazole and ivermectin in the second week. Praziquantel is used to treat schistosomiasis. Albendazole and ivermectin are available to children and adults due to ongoing treatment for lymphatic filariasis. Albendazole also is used to treat hookworm infections. The VCO instructed CMDs to approach all eligible individuals for all treatments within three weeks. This schedule was the national distribution schedule for praziquantel, albendazole, and ivermectin. However, the schedule was not necessarily the time limit for completion of drug distribution, as pills were not retrieved by the VCO from the study villages after three weeks. This practice is standard for MDA in Uganda. All CMDs were trained and instructed to begin distribution on October 1st, 2013. From October 1st-October 31st 2013, there was no interaction of CMDs with the VCO and researchers. No researchers were present for any training or distribution. One aspect of the training differed from routine MDA procedures. To ensure that all villages began distribution at the same time, the VCO was provided a car to reach all CMDs within the same time period for drug drop-off and training.

Participants and data sources

The following prompt was used to retrieve the number of household members to establish a household register.

> "How many people, including you, are in your household? The number of people in your household includes everyone who is under the same household head and can be in multiple homes. The household usually eats from the same pot. Do not include people who have been away from the village for more than six months. Only name people at least one year old."

A period of six months was used to ensure consistency in who was counted as a member across different households. Following the household prompt, the full surname (clan name), given name, nicknames, and previous names of each household member were recorded. The names were then directly inserted in later question prompts where information was collected on every household member.

Treatment coverage outcomes

The drug descriptions that accompanied the photos on the tablet computers were as follows.

> "This drug is praziquantel and is also called biltricide. It is used to treat bilharzia, which is an infection that you get from Lake Victoria. These are worms that live in your body and can cause enlarged liver and spleen. Victims may pass blood in their stool. The pill is a long white pill that tastes bitter and has a bad smell. The pill has three lines on it so it can be broken into four smaller pieces."
>
> "This drug is albendazole. It is used to treat gut worms, which you get from walking without shoes on the soil. The worms can cause diarrhea and abdominal pain. It is a long white pill. The pill is chewable and tastes like a sweet. The pill has one line on it so it can be broken into two smaller pieces."
>
> "This drug is ivermectin. It is used to treat elephantiasis, which is spread by mosquitoes and can cause extreme swelling of limbs. It is a very small round white pill. It has a mild taste like school chalk."

Amongst the initial 16,357 participants, 4.98% (815/16357) of people were ineligible for any treatments due to age (less than five years for praziquantel), pregnancy status (first trimester for ivermectin), or severe illness (ineligibility for all drugs). Only 4.82% (788/16357) of people could not be accounted for in the treatment population, of which 0.44% (72/16357) individuals were not asked about drug receipt due to a programming error in the tablet computers, 2.55% (417/16357) of individuals refused to answer, and 1.83% (299/16357) of participants were unsure about drug receipt. Therefore, 14,754 individuals were eligible for treatment. Amongst the eligible people, 99.13% (14625/14754) were in the main component of the village friendship networks (see Network Construction section) and were used to construct coverage outcomes.

The coverage outcomes concerned the reach and speed of treatment diffusion. Coverage is defined as the proportion of the eligible population that were offered treatment, i.e. visited and offered at least one drug by the CMD. Coverage was measured for eligible individuals in the 3,436 households belonging to the main components of the networks. There were 3,415 households with at least one person eligible for treatment. For individuals, coverage was a binary indicator that was equal to one if an individual was offered at least one of praziquantel, albendazole, or ivermectin by CMDs and was in the eligible population. Similarly, household coverage was a binary indicator equal to one if at least one eligible person in the home was offered at least one of the three MDA drugs by CMDs. Individual and household coverage indicators were then presented as proportions at the village level to assess the fraction of eligible individuals or households approached by CMDs for at least one drug. The day the drug was received was recorded for individuals who indicated being offered at least one drug by CMDs and was presented as a variable from one (offered a drug on the first day—October 1st—of distribution) to 31 (offered a drug on the last day—October 31st—of distribution). The earliest day of praziquantel, albendazole, or ivermectin receipt was used as a timestamp of drug receipt. There were 0.24% (35/14625) of eligible individuals who indicated receiving drugs greater than 31 days; these responses were not concentrated in one village. These answers were changed to the maximum response of 31 days. At the household level, the earliest day any eligible individual was offered praziquantel, albendazole, or ivermectin by CMDs was used as the timestamp of household drug receipt.

Household socioeconomic variables

All variables in this section were constructed at the household level. The following variables were presented as binary indicators. Social status represented if any adult in the household held (currently or previously) a position in the local village council or was a religious, tribe, or clan leader. The local village council in each village consisted of nine positions: chairman, vice chairman, secretary, defense, gender secretary, disabled secretary, youth council, information secretary, or elderly secretary. Binary indicators were used for if the household head was Muslim or belonged to the majority tribe of their village (Mudama or Musoga in the study region). The household was considered to purify water, an indication of preventative health behaviour, if they did any of the following activities after collecting drinking water: boil, add bleach, strain through cloth, use water filter, solar disinfect, wash jerry can with soap, or let stand and settle. The household had no home latrine if the respondent indicated that no facilities

were available and household members engaged in open defecation in a bush or field. Households that sought private medical care frequently received medical supplies and care from drug shops and private health clinics. Availability of electricity in the home also was recorded as a dummy variable.

Education was a count variable of the highest level of education attained by anyone in the household. Education was marked in levels from zero (no education) to 16. The levels of education were primary 1-7, senior 1-6 (levels 8-13), diploma (level 14), some university (level 15), and completed university (level 16). The years a household had lived in the village was represented as a continuous variable, rounded to the nearest year. Home quality score was a rank indicator of floor, wall, and roof materials. These materials were ranked from 1-4 and summed. The rank order was grass, sticks, plastic, and metal for the roof; mud and sticks, plastic, metal, and bricks or cement for the walls; and mud, plastic, wood planks, and brick or cement for the floor. Mud included cow dung. If no roof, wall, or floor material was present then zero was recorded.

Village-level variables

This section describes variables that measure village size, accessibility, and ecology. Two village size indicators were constructed. The total number of households included interviewed and not interviewed households in each village. There were a total of 3,578 households, which included 55 households that were not in the main component of the village friendship networks and 87 households that were not interviewed (see main text, network completeness). The fraction of total households that were friends with the CMDs also was calculated. Friendship was defined as a direct connection in the village social network (see network construction section). The physical size of the village was measured as follows. In November 2014, waypoints of all the homes in the village were taken. The physical homes in the village differed from the total households in the village, as some households lived in the same physical home but were counted as separate families in the household survey (see definitions in participants and data source section). There were 3,323 physical homes in the 17 study villages. In Python version 2.7.3 (www.python.org), the haversine distance in meters between each home and all other homes in the same village was calculated with Global Positioning System data. This procedure was repeated for every home in all the study villages. The haversine distance is the shortest distance (as-the-crow-flies) on the earth's surface between any two points. These distance matrices were used to calculate the average distance between any two homes in a village, which measured the general accessibility of the village for the CMDs [4]. The village ecology also had been surveyed in February 2013. This survey provided indirect measures of accessibility. Five binary indicators for village ecology were constructed. Three of these indicators assessed the presence of water bodies and were equal to one if the following were within the village: a large rice farm (rice paddy/swamp), beach on Lake Victoria, or small boat landing site on Lake Victoria (no beach). Other ecology dummy variables included if the village center was more than 0.50 kilometres from Lake Victoria and if there were three or more roads within a village. An additional village indicator was recorded. CMDs were asked if they used any methods not in the national protocol (i.e. other than door-to-door) to deliver treatments within their village. A binary indicator was constructed and equal to one if CMDs also made available an option for treatment pick-up from the CMDs' homes (the only other delivery option stated).

Network measurements

The friendship graphs were analyzed using Python version 2.7.3 (www.python.org) with several algorithms implemented from the NetworkX library [5]. Edges were not weighted and treated as undirected for all indicators except for reciprocity. Well-established centrality indicators [6] were measured. Degree is the total incoming and outgoing edges of a node and average neighbour degree is the mean degree of all neighbours of a node [6]. Reciprocated edges are counted as one edge for degree. Eigenvector centrality is similar to degree, but more weight is assigned to neighbours with more connections [6,7]. The eigenvector centrality of a node is proportional to the score of its neighbours. Hence, a node may have few connections, but have high eigenvector centrality because the neighbours of the node have high degree. Katz centrality captures not only the direct neighbours of a node, but also the extent that other nodes are connected to the node of interest through its neighbours. Katz is similar to eigenvector centrality except that there is a free parameter so all nodes have non-zero centrality. All connections to the node of interest contribute to its Katz centrality [6,8]. Three path-related centrality indicators for undirected edges were calculated: closeness, betweenness, and communicability. Closeness is the sum of shortest paths from a node to all other nodes in the network and is normalized by dividing by the sum of minimum distances [9]. Communicability is similar to closeness except this measure is not only concerned with the shortest paths, but also considers all paths connecting two nodes [10]. Betweenness centrality is the sum of the shortest paths between any two nodes in the

network that traverse the node of interest, which is divided by the total number of shortest paths [11]. Betweenness was normalized by 1/((n−1)(n−2)) where n is the total number of nodes in the network.

Three local measures of network transitivity were calculated—reciprocity, clustering, and density. In directed networks, reciprocity is the frequency that nodes received and returned connections. Reciprocity was measured as the total reciprocated edges divided by the total edges in the egocentric network of the CMD [6]. The egocentric network was a sub-graph of nodes and all edges between the CMD's household and its neighbours (direct connections) in the village network. Clustering was defined as the pattern of undirected edges that can exist between three nodes. Methods presented in Saramaki et al. were used to calculate clustering [12]. Clustering occurred when two connected nodes also shared connections with another node and was calculated as the fraction of possible triangles involving the node of interest and its neighbours. Local clustering was equal to the total number of connections between the neighbours of a node divided by the maximum possible number of connections between the neighbours. Clustering is important when comparing models of complex and simple contagion, and is being investigated in other fields including agricultural economics[13]. Density, in an undirected network, is equal to [2m/(n(n-1)] where m is the total number of edges and n is the total number of nodes [6]. Density was calculated for the egocentric networks of the CMDs.

Beyond centrality and local transitivity, two specific sub-graph network properties were examined [14,15]. The core number was calculated as described in Batagelj and Zaversnik [14]. The core number of a node is the value k where k is equal to the largest degree for all nodes in a maximal sub-graph. A k-core is a maximal sub-graph with all nodes that have at least degree k. Each vertex is connected to at least k other nodes in this sub-graph. A subset is maximal in that no single vertex can be added whilst maintaining the property of interest; here, the property is k connections amongst all nodes. If, any number of vertices may be added whilst retaining this property then the subset is not a k-core. The k-cores cannot overlap, as a group can only be a k-core if it is not a subset of a group that is a larger k-core. The clique number is the number of nodes in the largest maximal clique with the node of interest [15]. In a clique, every node is connected to every other node in the set. Cliques were allowed to overlap in the network. Since clustering was calculated with the immediate neighbours (friends) of a node, understanding the effects of the core and clique numbers provide insights into how well connected those friends must be. The core number enables a distinction between simply having a well-knit group of friends (clustering) or a highly connected group of friends who also share a high degree. This distinction is behaviourally interesting since high clustering, as opposed to a high core number, does not require a well connected, possibly influential group of friends. The clique number measures the completeness of connections between friends of a node. The significance of a high clique number for diffusion would indicate that the friends of a node or a subset of those friends must be perfectly connected. Understanding how complete connections must be amongst friends is needed to inform targets for public interventions that seek to introduce friends of a node.

Statistical analysis

Overview

To our knowledge, this study is the first analysis of complex networks for MDA [16]. All network variables except for reciprocity were calculated as undirected to assess who knows whom rather than who initiated contact. However, all analyses described below were repeated with directed network indicators except for clustering and density, which did not have a trivial directed measurement [6]. The direction and significance of all coefficients were robust and remained unchanged to the results found with undirected network variables. To identify the role of network indicators, the contribution of potential confounders was assessed, including homophily and village geography. With limited degrees of freedom (17 village observations) and, most importantly, collinearity between network indicators, univariate models were used for all statistical analysis. These models were robust to omitted variable bias (Supplementary Table 20).

Treatment coverage

To identify the topological indicators that predicted treatment coverage, network characteristics of CMDs were assessed in univariate fractional response models [17]. Unlike linear models, the magnitude of the coefficients of fractional response models cannot be directly interpreted. The expected margins of predictors found to be significant also were calculated to provide a more tangible measure of potential impact on the reach of treatment coverage. The dependent variables were the village outcomes (N=17) of household and individual coverage. Topological indicators were presented as the mean value for the two CMDs in each village. The average CMD network characteristics were then used as predictors for each of the dependent variables. These network characteristics included clustering, reciprocity in the CMD ego-network, density of the CMD ego-network, degree, average neighbour degree, core number, clique number closeness centrality, eigenvector centrality, katz centrality, betweenness centrality, and communicability. The fractional response models were specified with a probit link, binomial family, and robust standard errors [18]. To test for heteroskedasticity, the assumption of a fixed variance was relaxed and compared to models with fixed variance. If the equation for heteroskedasticity was significant (Wald test, p-value<0.05), the coefficient from the model with heteroskedasticity was reported and noted. As a robustness check, the treatment coverage models were examined as linear regressions and there was insufficient support to suggest any differences in the significance and sign of coefficients. However, unlike fractional response models, linear regressions cannot account for non-normal errors, non-linearity, heteroskedasticity (even with robust standard errors), and the bounded nature of the dependent variable when the model predictions (e.g. average partial effects) must lie between zero and one.

Treatment speed

To examine the speed of treatment distribution, CMD network properties were analyzed in univariate Poisson regressions with robust standard errors [18,19]. The speed of diffusion was measured by the time, in days, required for CMDs to not only approach but also have 50% of households accept or swallow treatment (effective coverage). Predicted margins were provided as an indication of the difference in speed for the 90th and 10th percentiles of significant variables, as the magnitudes of coefficients from Poisson regressions are not directly interpretable. Poisson regressions were used instead of proportional hazard models for the following reasons. In a proportional hazard model, if the baseline hazard is assumed to be constant over time than the proportional hazard model is the same as the Poisson model [20]. This relationship is due to the hazard estimator collapsing into a standard maximum likelihood estimator used in Poisson regression. Issues arise when attempting to use hazard models for our data. The sample size (15 villages that achieved 50% household coverage) is too small for this type of model and more equations are generated than actual observations. Hazard models also produced inconsistent predictions when there were 'ties' in the data, i.e. two villages achieving the target in the model on the same day.

The dependent variables for the speed of household and individual coverage were adjusted to account for missing data in the diffusion speed analysis. Individuals and households with missing day receipt information were excluded from the numerator and denominator (treated as missing data) when calculating individual and household coverage, respectively. The day of treatment offer was unavailable for 11.37% (944/8302) of eligible individuals who were visited by CMDs and refused treatment. And, not all individuals (3.98%; 293/7358), who accepted treatment when offered by CMDs, remembered the day of drug receipt. At the household level, the day of drug receipt was unavailable for 9.48% (228/2404) of households where everyone visited refused treatment, though these households can include unvisited individuals who might comply with treatment if offered by CMDs. And, there were 3.54% (85/2404) of households that were offered treatment and did not remember the day of drug receipt. The households that received treatment, but did not have any information for the day of drug receipt are presented by village in Supplementary Table 15. These households are roughly evenly spread across villages and were not a large proportion of CMD friends, except for Village ID 17 that was the smallest village with 65 households.

The predictors—the average CMD network characteristics—were the same as specified for the treatment coverage regressions. As the study was confined to one month (31 days), which was within the intended national schedule of drug distribution, not all villages reached 50% coverage and were included in the Poisson regressions. Fifteen villages achieved 50% household coverage whilst only 11 villages reached 50% individual coverage. The 50% coverage threshold was chosen to ensure comparability across villages, capture variability that was not present when achieving lower thresholds, avoid bias to small village size, and include the greatest number of villages without setting the threshold to a trivially low number. This 50% threshold is conservative for World Health Organization guidelines, which recommend 75% coverage as a feasible target [21]. A trivially low 20% target would have been needed to include all villages.

Robustness to temporal clustering, CMD friendship, and homophily

Temporal clustering, CMD friendship connections, and homophily were examined as potential confounders to the effect of CMD network properties on treatment diffusion. Univariate fractional response models with a probit link, binomial family, and robust standard errors were used when the dependent variable was household coverage [17,18]. For the speed of treatment as the dependent variable, univariate Poisson regressions with robust standard errors were used [18,19]. Temporal clustering was measured as the standard deviation of the day of drug receipt amongst friends of CMDs. This indicator excluded the CMDs. If significant, temporal clustering may suggest that any transitivity of connections amongst CMDs and their friends was confounded by the CMD simply approaching all friends at the same time. The standard deviation was calculated by using the earliest day that an eligible individual in the home was offered treatment by a CMD (see treatment outcomes section for day of drug receipt). Friendship status between CMDs was examined to understand the reliance of village treatment coverage on the connection between CMDs. A binary predictor was included and equal to one if the two CMDs were friends in the village network. The geodesic distance between CMDs in a village also was examined. Due to their initial selection, training, and working together for several years to administer treatments, all CMDs knew the other CMD within the same village irrespective of their close friendship status.

Homophily consists of the shared characteristics, environments, or other contextual factors amongst a group of individuals [22]. The presence of homophily can confound or, at the very least, inflate effects of network structure on diffusion [23]. The best practice for untangling the effect of homophily from other network properties involves experimental techniques [24]. Latent homophily, which is the set of shared and unobservable characteristics (for example, unrevealed attitudes or preferences), can only be addressed with a randomized controlled trial. Yet, this setup would undermine a key purpose of this study, which was to understand a practical context of diffusion where seed nodes are regularly selected by their fellow village members in a routine round of MDA. Accordingly, we have used the next best approach and directly examined all observable types of homophily, including manifest and secondary homophily [24]. Manifest homophily is a shared likeness on the characteristic of interest, which would suggest that CMDs acquired their friends because they were keen participants of MDA. Two indicators of the years of friendship were used to predict household coverage and speed of treatment. To assess if the CMD had acquired their friends after participating in MDA, a binary indicator was constructed and equal to one if the average years of friendship were less than the average years the CMDs had been active in MDA. If the CMDs joined after the start of MDA in Uganda (10 years at the time of study) then the average years active as a CMD may not be informative. Another binary variable measured if the average years of friendship were less than 10 years. We also assessed if the CMD personally had an affinity for MDA with the assumption that the length of tenure as a CMD may represent this affinity; the average years as a CMD also were used as an indicator of manifest homophily.

Secondary homophily is based on an observed characteristic that is not the outcome of interest. For example, CMDs may share traits with their friends that make CMDs more likely to better distribute drugs. To test this assumption, a wide range of household socioeconomic variables was used to predict household coverage and treatment speed. Household-level variables were used as CMD friendship connections were at this level. The construction of the following variables included the CMDs to measure the similarity amongst CMDs and their friends, although all analyses of these variables also were tested without the CMDs. The socioeconomic variables, if binary, were presented as the fraction of friends of the CMD that have the trait of interest. For count or continuous variables, the standard deviation of the trait amongst the friends was used as a predictor. The traits, as described in the socioeconomic variable section, included preference for private medical care, social status, Muslim household head, majority tribe, water purification, home latrine, private medical supply, education, years lived in the village, home quality score, and electricity in the home.

Robustness to village size, accessibility, and ecology

This section measured the general physical accessibility [4] of a village for a CMD. Accessibility was an important feature to study since CMDs were instructed to walk from home-to-home to distribute treatments. Accordingly, the total households, the fraction of total households connected to CMDs, and the average distance in meters between households were examined as predictors of household coverage and the speed at which 50% household coverage was reached. The univariate regressions were set up in the same manner as the treatment coverage and speed sections. Fractional response models with probit links and binomial families as well as Poisson regressions were used with robust standard errors [17-19], respectively, for the dependent variables of treatment coverage and speed. We

also sought to understand if the presence of swamps/rice paddies or fewer roads hinders CMDs from physically reaching a home, perhaps by blocking a footpath or simply requiring more time to reach the home. Additional ecological indicators, as described in the village-level variables section, that measured if there was a beach in the village, the distance of the village center to Lake Victoria, and the presence of a boat landing site were examined as possible predictors of treatment coverage and speed. In the event that other drug distribution methods facilitated treatment diffusion, an additional indicator of accessibility was examined that was unrelated to geography. This indicator tested the effect of CMDs allowing individuals to retrieve treatment from the CMDs' homes, which was in addition to home-to-home distribution and not part of the national training instructions.

Robustness to overfitting and variable selection

CMD clustering was selected as a predictor of treatment coverage when using several exploratory (not hypothesis-driven) methods. Such approaches complement our already presented and use of sparse models (univariate), forward selection, and factor analyses to reduce dimensionality. We employed data mining approaches to investigate model overfitting and variable selection, using *R*[25] with the glmnet[26] and hdm[27] packages. These approaches penalize the regression coefficients towards zero (shrinkage) and are useful for when the number of covariates exceeds the number of observations (high dimensionality). Three methods are available: ridge[28], lasso[29], and elastic net regressions[30]. Such methods are not hypothesis driven and do not consider the statistical significance of the variables. Ridge regression shrinks coefficient estimates, but retains all covariates and works well in the presence of correlated variables. This regression imposes a penalty on the coefficients that is equal to the square of the magnitude of the coefficient (L_2 regularization). Here, with ordinary least squares regression, ridge regression results in the shrinkage of the coefficient of average CMD clustering. Importantly, the penalized clustering (up to an unreasonably large penalty of $\lambda=10$) retains a value greater than zero (~0.01) against the dependent variable of household coverage[26]. Hence, CMD clustering remained relevant for positively predicting household coverage. On the other hand, lasso regression selects few variables and imposes a penalty based on the absolute value of the coefficient (L_1 regularization). However, this method performs poorly with correlated variables[30]. We observe this poor performance by implementing lasso with a data-driven penalty[31] and find no variables selected (including the intercept). No variables were selected, including the intercept, also in the case of double-post-lasso. When the lasso regression was rerun without the intercept, purely for variable selection as opposed to assessing an accurate model fit, we find CMD clustering (coef. 0.107) is amongst the five penalized variables selected (other variables were CMD education (0.152), water purification behaviour (0.036), core number (0.026), and clique number (0.009)). Lastly, we examined elastic net, which is a more flexible approach to shrinkage that does not make assumptions about the penalty. A mixing parameter, $\alpha[0,1]$, is included where either L_1 [$\alpha=1$] or L_2 [$\alpha=0$] or both regularizations can be employed. With $\alpha=0.40$, CMD clustering is selected at $\lambda=0.10$.

Focus group analyses

In April 2016, structured focus group questionnaires were developed at the Ministry of Health in Kampala and conducted in all of our study communities. Though the main aim of our study was to mitigate between two competing views, i.e. centrality versus clustering for seed node selection in social networks, the authors had an interest in further isolating the mechanism of the main result. To avoid biasing routine drug distribution, only an ex-post focus analysis was possible in our study. The researchers returned to the study area to meet with the drug distributors and chairmen (village leaders) of all the 17 study villages. Individuals were directly asked: "Is there any benefit of having a close-knit group of friends to deliver more treatments during mass drug administration?" All participants enthusiastically agreed. The main finding in all villages and corroborated by quotes from the focus groups, is that close-knit friends feed back information to the CMDs about problems in the community and about missed households. One chairman and CMD explained that "communication is much easier and they can talk to each other about the problems that they are facing. [Friends] tend to meet each other when everyone is connected; when they are [a] close group they can be together." Other CMDs indicated that receiving the same information from close-knit friends assisted treatment distribution: "For them, they can reach many people because they are sharing the same information."

The secondary finding of the focus groups in all villages was that close-knit friends increased the performance of CMDs by spreading information to the wider community and mobilizing households. For example, one CMD stated that close-knit friends "can help them do work quicker. [Close-knit friends] helps (sic) to give information back to the person distributing. [And, a] certain household will already be informed about drug distribution." Another CMD noted that "[I] can tell my friends to go around and mobilize people and they come quickly." Concerning

mobilization of the wider community, all CMDs expressed concern that information about drug availability often needs to be provided repeatedly to the same recipients. These results suggest that social reinforcement occurs through the process of spreading repeated information not only to the wider community (complex contagion), but more importantly feeding back information to the CMD. Our qualitative evidence suggests that clustering facilitates this type of repeated information exchange.

Supplementary Figure 1 Village degree distributions

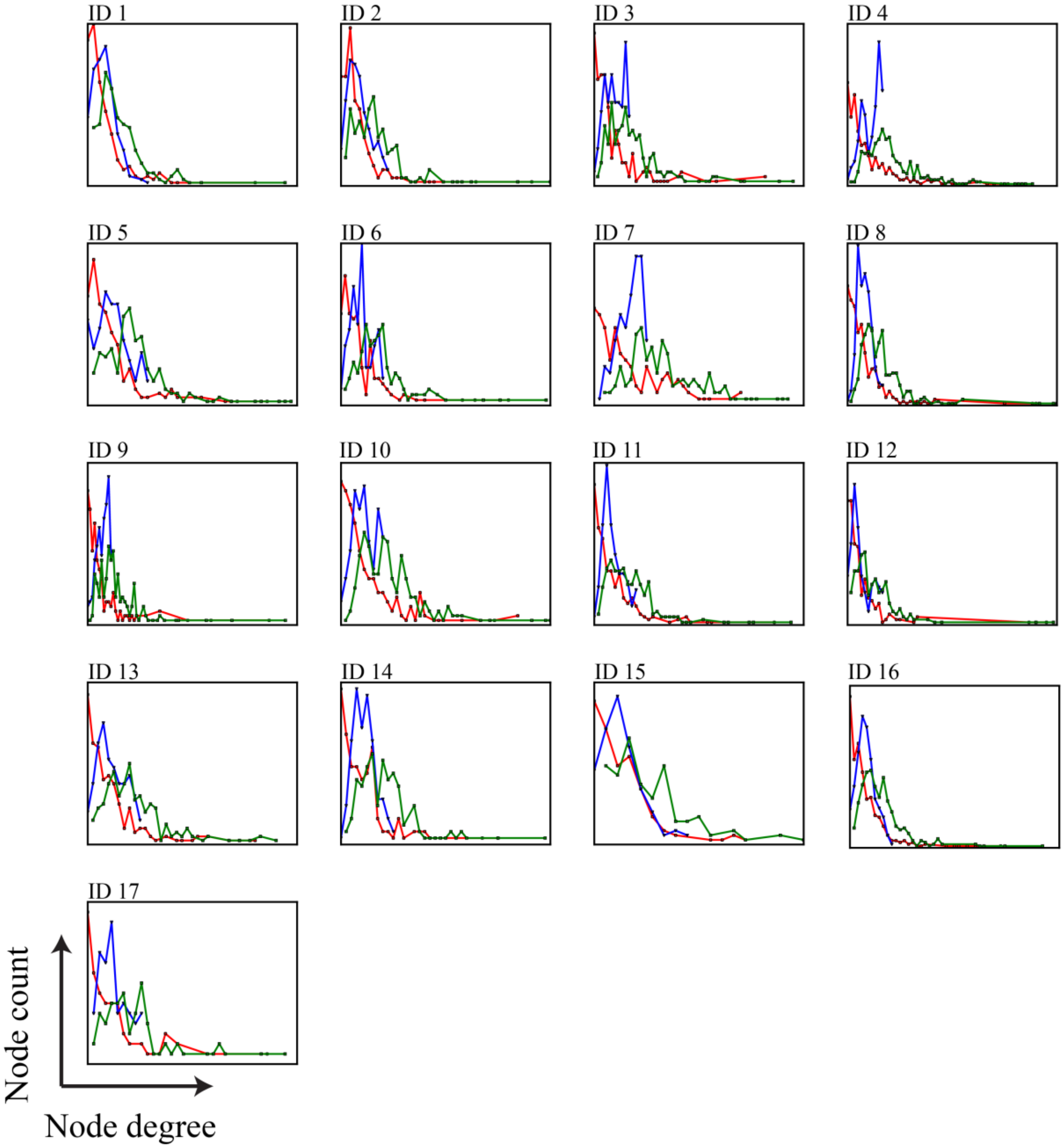

The linear probability density functions are plotted. The number of each plot corresponds to the village ID and the plots are not ordered by any village characteristics. In-degree, out-degree, and degree are plotted respectively in red, blue, and green. The y-axis represents the frequency of nodes and the x-axis is the actual node degree, in-degree, or out-degree. The frequency counts are not binned; the raw counts and every occurring degree, in-degree, or out-degree in a village is displayed. All villages displayed right-skewed degree distributions where only a small number of nodes have many connections.

Supplementary Figure 2 Correlation of avg. community medicine distributor and global network characteristics

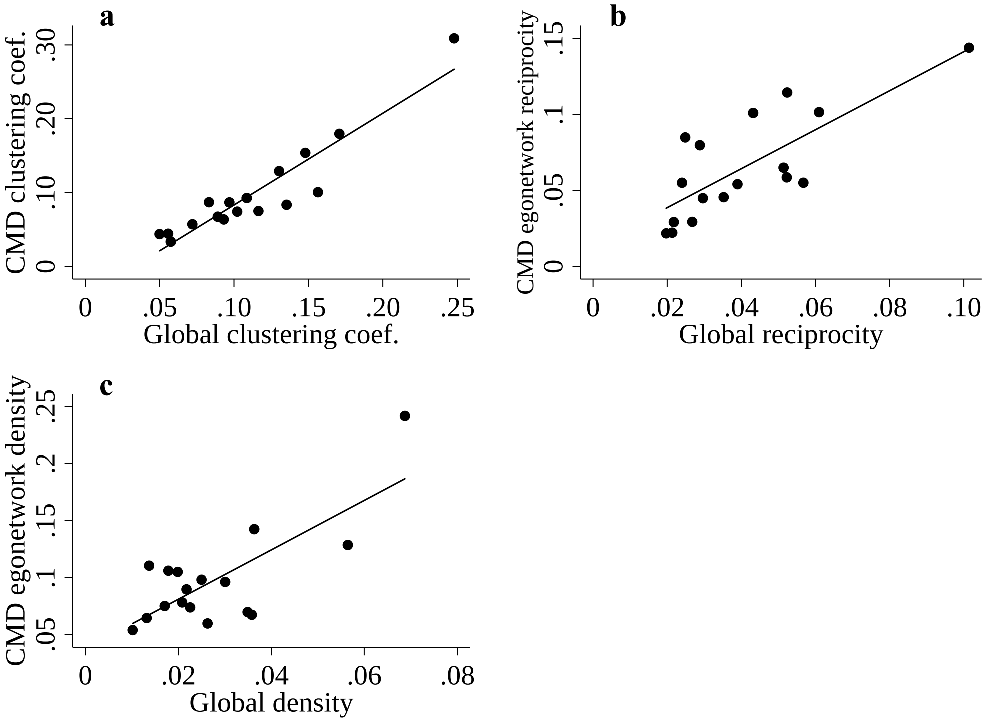

Supplementary Figure 2 presents the relationship of local transitivity around the community medicine distributors (CMDs) with global network transitivity. The global characteristics are calculated for the village network, whereas the CMD characteristics represent the average of local CMD properties. A simple linear fit is projected on the scatter plots, though this line does not necessarily represent a significant relationship. **a)** The correlation of CMD clustering and network clustering (calculated as the number of triangles occurring in the network out of the maximum possible triangles) was significant (Spearman coeff. 0.8873; p-value<0.0001). **b)** Average reciprocity of the CMD ego-networks was positively associated with the average village network reciprocity (Spearman coeff. 0.7468; p-value=0.0006). **c)** Average density of CMD ego-networks was not significantly associated with density of the full village network (Spearman coeff. 0.3946; p-value=0.1170).

Supplementary Figure 3 Time series of individual treatment coverage by village

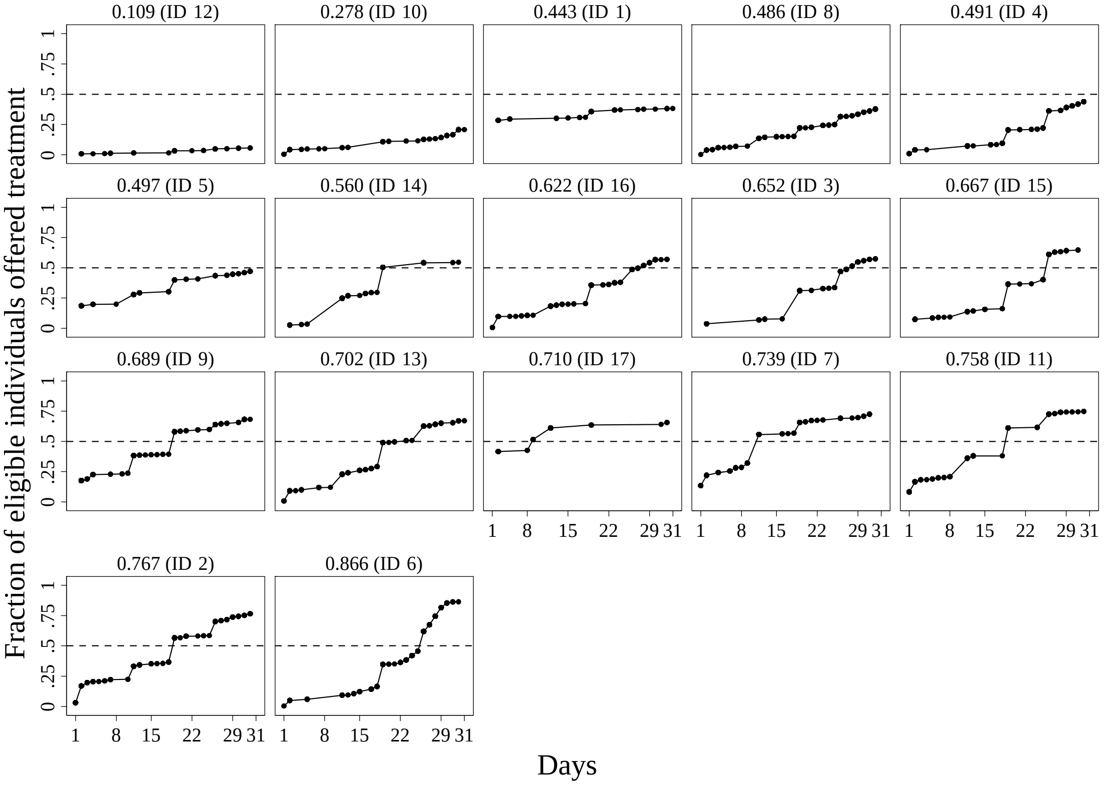

Each plot represents the fraction of eligible individuals who were offered treatment by a particular day of the distribution period. The day of drug receipt was unavailable for individuals refusing treatment and these individuals were excluded from the numerator and denominator when calculating the fraction of eligible people offered treatment. To enable comparisons with the reach of diffusion, the graphs are ordered by total individual coverage achieved, which is stated in the plot title. Total individual coverage is the proportion of eligible individuals in the village who were offered at least one drug of praziquantel, albendazole, or ivermectin from a community medicine distributor. The total individual coverage may differ than the graph maxima as total individual coverage includes noncompliers and individuals who did not remember the day of drug receipt. The village ID is provided in parentheses. The dashed line represents 50% individual treatment. Village ID 17, which had a shorter distribution span than the other villages, was the smallest village of only 65 households.

Supplementary Figure 4 Map of study villages and households

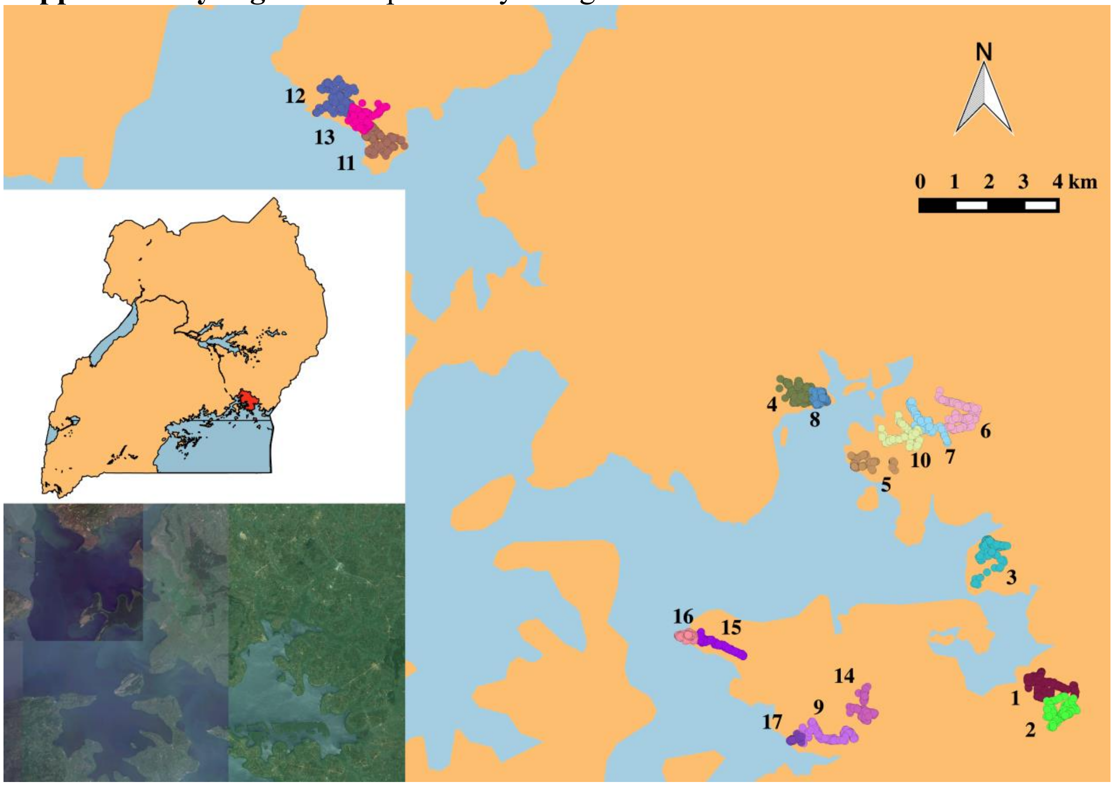

Supplementary Figure 4 presents a map of the study villages and households within those villages. The two inserts show the location of the study district (Mayuge) in Uganda and the satellite imagery of the study area terrain. The satellite data (Landsat images) are distributed by the Land Processes Distributed Active Archive Center (LP DAAC), located at USGS/EROS, Sioux Falls, SD. http://lpdaac.usgs.gov.[32] The numbers next to the villages correspond to the village IDs used in the main text. From this figure, a wide variation in village 'shape' is observable, as some villages have households that are geographically clustered, whereas other villages have households that are spread out. No study villages were isolated from other villages in the study area, i.e. all villages had geographical neighbours including Village ID 3. Villages that were isolated included villages in the land area between village ID 11 and 4; these villages that were not included in our study were surrounded by government forests, were nearly a kilometer from neighbouring villages, and were difficult to access by even informal dirt roads. The villages that achieved the highest household coverage (IDs 6, 7, 15, 17, 13) and the fastest speed of household coverage (IDs 17, 9, 7, 1, 3) did not all belong to the same geographical cluster in Supplementary Figure 4.

Supplementary Figure 5 Photos of drugs distributed through mass drug administration

Praziquantel Albendazole Ivermectin

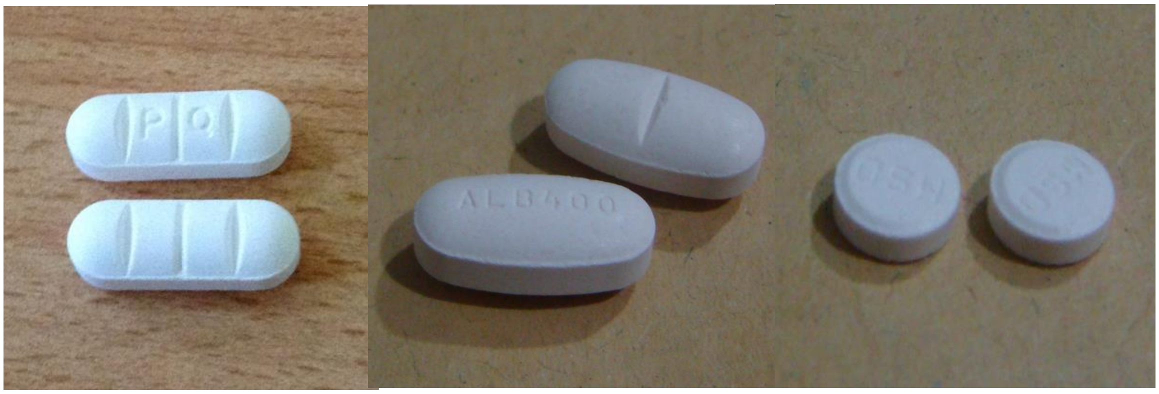

Supplementary Table 1 Network generation and global properties by village

	A) Network generation				*B) Network characteristics*					
**Vill. ID**	**Nodes** (House -holds)	**Households interviewed** (Max possible nodes)	**Self-loops removed**	**Edges**	**Mean degree**	**Mean in-degree**	**Mean geodesic distance** (undirected)	**Mean geodesic distance** (directed)	**Exponent (alpha) of power law distribution**	**Clustering coefficient** (undirected)
1	202	212	21	558	5.416	2.762	3.224	2.447	1.680	0.057
2	181	185	19	709	7.602	3.917	2.759	3.012	1.555	0.109
3	192	193	16	1103	11.042	5.745	2.444	2.768	1.454	0.130
4	320	320	39	2127	12.975	6.647	2.533	2.869	1.418	0.089
5	184	191	8	759	7.826	4.125	2.802	2.580	1.534	0.116
6	139	139	8	670	9.094	4.820	2.488	3.088	1.485	0.135
7	121	121	18	820	12.727	6.777	2.158	2.363	1.408	0.171
8	369	369	28	1792	9.501	4.856	2.797	3.162	1.478	0.056
9	178	180	10	1129	12.022	6.343	2.329	2.474	1.432	0.156
10	207	209	13	1122	10.551	5.420	2.540	2.828	1.455	0.102
11	250	255	12	1111	8.504	4.444	2.794	2.748	1.510	0.093
12	229	229	13	891	7.616	3.891	2.881	3.144	1.564	0.083
13	183	184	5	833	8.842	4.552	2.622	2.810	1.506	0.097
14	124	128	13	554	8.468	4.468	2.516	2.659	1.502	0.148
15	120	133	5	284	4.567	2.367	3.274	2.006	1.766	0.072
16	372	378	7	1407	7.376	3.782	3.127	3.097	1.554	0.050
17	65	65	2	286	7.908	4.400	2.226	2.142	1.522	0.248

Supplementary Table 1 presents the basic network characteristics of each village friendship network. These networks were unweighted and both directed and undirected versions were utilized for calculating global network statistics. Although global network features are not the focus of this paper, a summary of the village network characteristics is provided in Supplementary Table 1. These global characteristics demonstrate that the 17 study villages displayed properties commonly found in real-world networks [33]. Three global network statistics are included in Supplementary Table 1 that were not described in the materials and methods section. The mean geodesic distance is the average of all shortest paths between two nodes in the network. We provide both directed and undirected measures of average geodesic distance and also degree. In a directed network, there may be no paths between two nodes and these ‘infinite’ paths are not counted in the average shortest path. The directed measure of degree provided is in-degree, which is the count of incoming connections. Households were allowed to name up to ten outgoing connections and average degree may differ from average in-degree due to the count of these outgoing connections.

The exponent of the power law distribution, alpha, was calculated using methods described in Alstott et al. [34]. We assessed if a power law degree distribution was a better fit (log-likelihood test) when compared to an exponential or lognormal distribution. All nodes in the main component, i.e. at least one degree, were considered and degree was treated as a continuous variable. A power-law distribution was a better fit than both exponential and lognormal distributions in all villages with all p-values<0.001. Global clustering was defined as the total triangles that occurred in the network divided by the maximum number of triangles theoretically possible.

Supplementary Table 2 Network statistics of community medicine distributor (CMD)

Variable	Obs.	Mean	Std. Dev.	Min	Max
Clustering	34	0.099	0.075	0	0.429
Reciprocity of CMD ego-network	34	0.065	0.040	0	0.176
Density of CMD ego-network	34	0.098	0.050	0.033	0.321
Degree	34	28.029	15.762	7	80
Avg. neighbour degree	34	12.069	3.102	6.067	17.469
Core number	34	6.176	1.445	3	9
Clique number	34	4.118	0.880	2	6
Closeness	34	0.470	0.070	0.331	0.641
Eigenvector	34	0.183	0.075	0.045	0.354
Katz	34	0.198	0.099	0.001	0.374
Betweenness	34	0.061	0.052	0.005	0.229
Communicability	34	0.316	0.207	0.026	0.887

Supplementary Table 2 provides a summary of the network characteristics for the households of CMDs. With an average degree of over 30, CMDs have more than double the average degree of the village network (Supplementary Table 1). No limits were placed on degree or in-degree, so the natural limit for both indicators was the total number of nodes in the village minus one (considering self-loops and multi-edges were ignored). No one in a village reached this limit. The maximum degree in a village ranged from 17-80. In 35.29% (6/17) of villages, community medicine distributors had the highest value of degree (though not necessarily exclusively). The centrality of CMDs can be expected to be higher than the average centrality in the village. These individuals were elected to be CMDs by other village members.

Supplementary Table 3 Summary of coverage outcomes and descriptive statistics for CMDs and friends

	A) CMDs' households			B) CMDs' friends' households		
**Descriptive variables**	**Obs.**	**Mean** *Proportion*	**Std. Dev.** *Frequency*	**Obs.**	**Mean** *Proportion*	**Std. Dev.** *Frequency*
Household coverage [a]	34	*0.9118*	*31/34*	809	*0.7305*	*591/809*
Individual coverage [b]	254	*0.7835*	*199/254*	3957	*0.6002*	*2375/3957*
Earliest day of drug offer for an eligible household member (within the one month drug distribution) [c]	31	9.903	8.972	538	12.262	9.073
Social status: at least one household member was or is a religious, tribe, or clan leader or on the local village council	34	*0.4412*	*15/34*	811	*0.1332*	*108/811*
Muslim household head	34	*0.2647*	*9/34*	811	*0.3243*	*263/811*
Household head belongs to majority tribe	34	*0.4706*	*16/34*	796	*0.4598*	*366/796*
Household purifies drinking water	34	*0.6765*	*23/34*	811	*0.4427*	*359/811*
No home latrine	34	*0*	*0/34*	811	*0.0678*	*55/811*
Household seeks medical care from private clinics	34	*0.7353*	*25/34*	811	*0.5845*	*474/811*
Highest level of education attained by a household member	34	10.059	2.828	808	7.254	3.059
Total years household settled in village	34	18.059	10.685	810	15.795	12.078
Home quality score	34	8.618	3.153	811	7.189	3.347
Household electricity	34	*0.1176*	*4/34*	811	*0.0752*	*61/811*
Years active as CMD [d]	32	7.594	3.359	-	-	-

[a] Household coverage is defined as the proportion of households in the village where at least one eligible person in the home was offered at least one treatment through mass drug administration.
[b] Individual coverage is defined as the proportion of eligible individuals in the village who were offered at least one treatment through mass drug administration.
[c] Excludes the actual CMD.
[d] CMDs were asked the number of years that they have been a CMD, which ranged from 1 (first year as CMD) to 10 (all years MDA had been ongoing in Uganda). Two of 34 CMDs (Village ID 3 and 17) did not provide the number of years they have been active as a CMD.

Supplementary Table 3 presents the socio-demographic statistics for the community medicine distributors (CMDs) and the friends of the CMDs. When compared to their friends and to the average of all households in the study area (see Supplementary Table 4), the CMDs had higher social status, better home quality, higher educational attainment, more access to formal medical care, lived in the village longer, all had a home latrine, and more purified drinking water. Hence, the CMDs not only had high centrality, but also high socio-demographic status. Again, this finding was not surprising as villagers elect the CMDs. CMDs also offered treatment to more individuals in their home, on average, when compared to the rest of the village. Individual coverage may appear lower than household coverage for CMDs, but 26/55 people not offered treatment in CMDs’ homes were all from one village where both CMDs did not offer treatment to anyone in their homes (Village ID 12).

Supplementary Table 4 Summary of coverage outcomes and descriptive statistics for all households

	A) All households in friendship networks, excluding CMDs[a] and their friends			*B) All households in friendship networks*		
**Descriptive variables**	**Obs.**	**Mean** *Proportion*	**Std. Dev.** *Frequency*	**Obs.**	**Mean** *Proportion*	**Std. Dev.** *Frequency*
Household coverage [b]	2572	*0.6928*	*1782/2572*	3415	*0.7040*	*2404/3415*
Individual coverage [c]	10414	*0.5500*	*5728/10414*	14625	*0.5677*	*8302/14625*
Earliest day of drug offer for an eligible household member (within the one month drug distribution) [d]	1522	13.286	9.077	2091	12.972	9.089
Social status: at least one household member was or is a religious, tribe, or clan leader or on the local village council	2591	*0.0664*	*172/2591*	3436	*0.0859*	*295/3436*
Muslim household head	2591	*0.2787*	*722/2591*	3436	*0.2893*	*994/3436*
Household head belongs to majority tribe	2550	*0.3682*	*939/2550*	3380	*0.3914*	*1323/3380*
Household purifies drinking water	2591	*0.3840*	*995/2591*	3436	*0.4008*	*1377/3436*
No home latrine	2591	*0.1104*	*286/2591*	3436	*0.0992*	*341/3436*
Household seeks medical care from private clinics	2591	*0.6110*	*1583/2591*	3436	*0.6059*	*2082/3436*
Highest level of education attained by a household member	2586	6.787	2.890	3428	6.93	2.953
Total years household settled in village	2588	12.991	10.724	3432	13.703	11.127
Home quality score	2591	6.298	3.405	3436	6.531	3.416
Household electricity	2591	*0.0533*	*138/2591*	3436	*0.0591*	*203/3436*

**Village-level variables**	**Obs.**	**Mean** *Proportion*	**Std. Dev.** *Frequency*	**Min**	**Max**
Household coverage	17	0.7255	0.1751	0.2314	0.9353
Individual coverage	17	0.5905	0.1913	0.1093	0.8658
Total households in village	17	210.471	88.488	66	395
Fraction of total households connected to CMDs	17	0.265	0.131	0.109	0.594
Rice paddy (large rice farm) within village	17	*0.7647*	*13/17*	-	-
Beach on Lake Victoria within village	17	*0.4118*	*7/17*	-	-
Lake site only within village (no beach, but small boat landing site) on Lake Victoria	17	*0.3529*	*6/17*	-	-
Village centre more than 0.50 km to Lake Victoria	17	*0.5882*	*10/17*	-	-
3 or more roads within village	17	*0.4706*	*8/17*	-	-
Average metres between two households in the same village	17	400.114	142.273	130.613	638.669

[a] Community medicine distributor.
[b] Household coverage is defined as the proportion of households in the village where at least one eligible person in the home was offered at least one treatment through mass drug administration.
[c] Individual coverage is defined as the proportion of eligible individuals in the village who were offered at least one treatment through mass drug administration.
[d] Excludes the actual CMD.

Supplementary Table 4 presents basic household socio-demographic variables of the study population and village-level outcomes and covariates. The socio-demographic statistics are provided for all households and the households that exclude CMDs and their friends to enable comparisons with Supplementary Table 3. Amongst all eligible individuals, 37.55% (5491/14625) of people had not received any information that MDA was ongoing. Amongst only eligible individuals who were not offered treatment, 86.64% (5491/6323) of individuals had not heard MDA was ongoing during the one-month distribution period.

Supplementary Table 5 Global network transitivity against coverage achieved and speed of treatment

Univariate regressions[a]	A) Dependent variable: Household coverage[b]					B) Dependent variable: Individual coverage[c]				
**Global network transitivity variables**	**Coeff.**	**Robust Std. Err.**	**p-value**	**95% CI**		**Coeff.**	**Robust Std. Err.**	**p-value**	**95% CI**	
**Treatment Coverage**[d]										
Clustering	3.575	1.646	0.030	0.349	6.801	3.902	1.478	0.008	1.005	6.800
	Obs. 17					Obs. 17				
**Speed of Treatment**[e]										
Clustering	-5.475	2.444	0.025	-10.264	-0.685	-5.334	1.123	<0.001	-7.534	-3.133
Reciprocity	-15.423	6.461	0.017	-28.087	-2.759	-14.165	3.481	<0.001	-20.986	-7.343
Density	-19.376	8.129	0.017	-35.309	-3.442	-17.574	3.618	<0.001	-24.664	-10.483
	Obs. 15 [f]					Obs. 11 [f]				

[a] Single predictor regressions.
[b] Household coverage is defined as the proportion of households in the village where at least one eligible person in the home was offered at least one treatment through mass drug administration.
[c] Individual coverage is defined as the proportion of eligible individuals in the village who were offered at least one treatment through mass drug administration.
[d] Univariate fractional response regressions were used with probit link; constants not shown. The dependent variables were the fraction of eligible households or individuals who were offered treatment in the village.
[e] Univariate Poisson regressions were used; constants not shown. The dependent variables were the speed of treatment (number of days out of one-month distribution until community medicine distributors offered treatment to 50% households or individuals).
[f] Only 15/17 and 11/17 villages reached, respectively, 50% household or 50% individual coverage within the one-month distribution period that was studied.

Supplementary Table 5 presents the relationship of global network transitivity with outcomes of treatment coverage and speed of treatment. The network variables presented are the variables that were found to be significant in the main text Tables 1-2. Supplementary Table 5 is a robustness check for the main text analysis; particularly as average network properties of community medicine distributors (CMDs) were associated with global network properties (Supplementary Figure 2). Each network characteristic was calculated for the whole network in the same manner as described in the methods for node-level (CMD) calculations. The significance and direction that was found for the average of CMD network properties was preserved when these properties were examined at the village level.

Supplementary Table 6 Clustering and centrality correlation table

**Average CMD network properties[a]**	Clustering	Reciprocity of CMD ego-network	Density of CMD ego-network	Degree	Avg. neighbour degree	Core number	Clique number	Closeness	Eigenvector	Katz	Between-ness	Commun-icability
Clustering	1.000											
Reciprocity of CMD ego-network	0.730*	1.000										
Density of CMD ego-network	0.862*	0.720*	1.000									
Degree	-0.092	-0.240	-0.509*	1.000								
Avg. neighbour degree	0.253	0.096	-0.108	0.457	1.000							
Core number	0.203	0.060	-0.141	0.507*	0.965*	1.000						
Clique number	0.414	0.136	-0.059	0.806*	0.635*	0.620*	1.000					
Closeness	0.479	0.194	0.032	0.760*	0.571*	0.591*	0.931*	1.000				
Eigenvector	0.093	-0.063	-0.258	0.776*	0.035	0.061	0.691*	0.746*	1.000			
Katz	0.112	0.008	-0.252	0.777*	0.132	0.153	0.744*	0.775*	0.951*	1.000		
Betweenness	-0.015	-0.234	-0.243	0.681*	-0.162	-0.094	0.443	0.555*	0.863*	0.700*	1.000	
Communicability	0.183	-0.049	-0.239	0.899*	0.349	0.386	0.863*	0.914*	0.922*	0.922*	0.770*	1.000

Obs. 17
* p-value<0.05
[a]Community medicine distributor. The average of network properties for the two CMDs in each village were used in pairwise correlations.

Clustering was not associated (p-value>0.05) with any centrality indicators. However, clustering was associated (p-value<0.05) with other measures of local transitivity, including CMD ego-network density and reciprocity.

Supplementary Table 7 Descriptive properties of frequency distributions for day of drug receipt

	A) Distribution of day of household treatment [a]							B) Distribution of day of individual treatment [b]						
Vill ID	Obs.[a]	Mean	Std. Dev.	Skew-ness	p-value	Kurt -osis	p-value	Obs.[b]	Mean	Std. Dev.	Skew - ness	p-value	Kur-tosis	p-value
1	113	5.212	7.888	1.630	<0.001	4.365	0.017	286	5.077	7.653	1.601	<0.001	4.197	0.003
2	146	11.562	8.661	0.089	0.648	1.718	<0.001	619	13.341	9.096	-0.076	0.438	1.770	<0.001
3	109	17.541	7.584	-0.713	0.003	3.012	0.736	401	19.107	6.759	-1.030	<0.001	4.010	0.002
4	175	16.343	8.876	-0.544	0.004	2.207	0.001	505	19.289	7.891	-0.986	<0.001	3.334	0.131
5	101	7.634	8.425	1.004	<0.001	2.903	0.925	388	10.245	9.133	0.499	<0.001	2.063	<0.001
6	128	19.516	7.161	-1.255	<0.001	4.000	0.041	636	20.068	6.934	-1.237	<0.001	4.111	<0.001
7	100	8.350	7.872	0.900	0.001	3.188	0.480	410	8.841	7.606	0.736	<0.001	3.094	0.578
8	195	14.533	8.660	-0.082	0.628	1.928	<0.001	455	16.240	8.900	-0.252	0.028	1.890	<0.001
9	136	8.757	7.915	0.707	0.001	2.558	0.267	575	11.346	8.499	0.265	0.010	2.031	<0.001
10	62	16.726	11.880	-0.275	0.339	1.456	<0.001	151	18.106	10.909	-0.481	0.012	1.785	<0.001
11	190	12.137	8.405	-0.079	0.645	1.872	<0.001	658	12.375	8.362	-0.094	0.323	1.858	<0.001
12	31	16.645	9.386	-0.421	0.275	1.931	0.065	58	17.103	8.757	-0.613	0.048	2.253	0.143
13	135	13.696	7.668	-0.123	0.541	2.266	0.012	526	14.930	7.960	-0.284	0.008	2.289	<0.001
14	89	13.022	5.488	-0.113	0.641	3.505	0.222	248	13.492	5.352	0.009	0.950	3.475	0.127
15	93	14.538	8.472	-0.458	0.064	1.900	<0.001	367	17.275	7.781	-0.928	<0.001	2.794	0.463
16	247	14.494	8.782	-0.253	0.100	1.868	<0.001	651	15.860	8.779	-0.457	<0.001	2.035	<0.001
17	41	3.610	4.471	1.568	<0.001	4.701	0.033	131	4.664	6.245	2.265	<0.001	8.843	<0.001

[a] Households where at least one eligible person was offered treatment and the day of drug receipt was known.
[b] Individuals who were offered at least one drug and the day of drug receipt was known.

The frequency of households or individuals being offered treatment at each day of the one-month distribution period was plotted for each village and descriptive statistics of those frequency distributions are presented in Supplementary Table 7. Skewness measures the symmetry of the frequency distributions whereas kurtosis measures the thickness of the distribution tails. More specifically for skewness, if the median is greater than the mean, the value for skewness will be negative and the distribution will be left-skewed. Kurtosis describes if the peak of the distribution is flat or pronounced. These measures were calculated as described in D'Agostino et al.[35] For comparison, Normal curves have skewness and kurtosis of approximately zero and three, respectively. The p-values provided are for a chi-squared test of skewness and kurtosis against values expected in a Normal distribution, i.e. the null hypothesis is that the distribution is a Normal curve. There was no trend in the skewness of the distributions for the villages that achieved the highest household or individual coverage. For example, the top five villages for household coverage (IDs 6, 7, 15, 17, 13) had varied distributions that were either more left-skewed, right-skewed, or insignificantly different from a Normal distribution. The top five fastest villages (IDs 17, 9, 7, 1, 3) that achieved 50% household coverage in the fewest days had four villages with right-skewed distributions (IDs, 17, 9, 7, 1) when compared to Normal curves. However, the third (ID 5) and fifth (ID 1) slowest villages also had significantly (p-value<0.001) right-skewed distributions when compared to Normal curves. As per kurtosis, there was no notable trend in the tails of distributions for the best performing villages. The villages with the fastest or highest coverage had varied kurtosis that was higher, lower, or insignificantly different than Normal curves. Overall, there was no apparent difference in the skewness or kurtosis between the fastest and slowest villages, so differences in how the CMDs approached households was not easily observable in the distribution of days in which treatment was offered.

Supplementary Table 8 Coverage and speed of treatment for individual coverage

Univariate regressions	*A) Dependent variable: Individual coverage[a]*					*B) Dependent variable: Number of days to reach 50% individual coverage[b]*				
**Average CMD[c] network properties**	**Coeff.**	**Robust Std. Err.**	**p-value**	**95% CI**		**Coeff.**	**Robust Std. Err.**	**p-value**	**95% CI**	
Clustering	2.053	0.758	0.007	0.567	3.539	-4.418	0.788	<0.001	-5.962	-2.874
Reciprocity of CMD ego-network[d]	77.03	51.146	0.132	-23.213	177.274	-7.815	2.739	0.004	-13.183	-2.447
Density of CMD ego-network	3.164	1.757	0.072	-0.279	6.607	-6.296	1.113	<0.001	-8.477	-4.116
Degree	-0.004	0.008	0.628	-0.020	0.012	0.003	0.005	0.558	-0.007	0.014
Avg. neighbour degree	-0.004	0.027	0.871	-0.056	0.048	-0.025	0.039	0.513	-0.101	0.05
Core number	0.009	0.052	0.868	-0.093	0.111	-0.058	0.072	0.416	-0.198	0.082
Clique number	0.07	0.107	0.511	-0.139	0.279	-0.082	0.086	0.339	-0.25	0.086
Closeness	1.274	1.33	0.338	-1.333	3.881	-1.429	1.187	0.229	-3.755	0.898
Eigenvector	0.379	1.733	0.827	-3.018	3.775	1.015	1.149	0.377	-1.237	3.267
Katz	0.615	1.237	0.619	-1.81	3.04	0.084	1.062	0.937	-1.997	2.165
Betweenness	-1.717	4.02	0.669	-9.596	6.162	1.92	2.014	0.341	-2.028	5.868
Communicability	0.112	0.587	0.849	-1.039	1.263	-0.03	0.336	0.929	-0.689	0.629
	Obs. 17					Obs. 11[e]				

[a] Each model is a single predictor regression. Fractional response models were used with probit link; constants are not shown. Individual coverage is defined as the proportion of eligible individuals in the village who were offered at least one treatment through mass drug administration.
[b] Each model is a single predictor regression. Poisson regressions were used; constants are not shown. The speed of treatment was the number of days within a one-month distribution period until 50% of eligible individuals were offered at least one treatment through mass drug administration.
[c] Community medicine distributor. The average of network properties for the two CMDs in each village were used as predictors in the univariate regressions.
[d]Adjusted for heteroskedasticity in the fractional response models of individual coverage.
[d]Only 11/17 villages achieved 50% individual coverage within one-month of mass drug administration.

Supplementary Table 8 presents the association of CMD network properties with the reach and speed of treatment diffusion for eligible individuals. **Panel A.** The results for individual coverage are consistent with the results for household coverage presented in the main text Table 1. **Panel B**. Similar to Panel A, Panel B is presented as a robustness and consistency check for the speed of household coverage in the main text Table 2. All results found in the main text remained robust when the proportion of eligible individuals as opposed to households was used as the dependent variable.

Supplementary Table 9 Robustness of treatment coverage to temporal effects, CMD friendship, and homophily

	**Univariate regressions[a]**	***Dependent variable: Household coverage[b]***				
	**Predictors**	**Coeff.**	**Robust Std. Err.**	**p-value**	**95% CI**	
**A) Temporal effects & CMD[c] friendship**	Std. Dev. of day of drug receipt for households that are friends with CMDs (Temporal clustering, excluding CMDs)	-0.075	0.057	0.188	-0.186	0.037
	CMDs are directly connected in friendship network	-0.510	0.288	0.077	-1.075	0.055
**B) Manifest homophily**	Shortest path (geodesic distance) between CMDs in friendship network	0.157	0.185	0.397	-0.206	0.519
	Average years as CMD is less than average years of friendship amongst friends of CMDs	-0.243	0.192	0.206	-0.620	0.134
	Average years of friendship amongst friends of CMDs is less than MDA existence (10 years)	-0.025	0.322	0.938	-0.657	0.606
	Average years as CMD	0.037	0.048	0.441	-0.057	0.130
**C) Secondary homophily**	Household seeks medical care from private clinics[d]	0.522	0.536	0.330	-0.528	1.572
	Social status: at least one household member was or is a religious, tribe, or clan leader or on the local village council[d]	2.384	1.293	0.065	-0.150	4.919
	Muslim household head[d]	-1.772	1.202	0.141	-4.128	0.585
	Household head belongs to majority tribe[d]	-1.480	1.508	0.326	-4.435	1.476
	Household purifies drinking water[d]	-0.541	0.662	0.413	-1.838	0.755
	Home latrine[d]	1.153	1.395	0.409	-1.582	3.887
	Highest level of education attained by a household member[e]	0.022	0.200	0.913	-0.370	0.414
	Total years household settled in village[e]	-0.076	0.065	0.243	-0.204	0.052
	Home quality score[e]	-0.138	0.238	0.563	-0.603	0.328
	Household had electricity[d]	-2.940	2.158	0.173	-7.169	1.289

Obs. 17

[a] Each model is a single predictor regression. Fractional response models were used with probit links; constants are not shown.
[b] Household coverage is defined as the proportion of households in the village where at least one eligible person in the home was offered at least one treatment through mass drug administration.
[c]CMD is an abbreviation for community medicine distributor.
[d]Predictors were represented as the percentage of CMDs friends (including the CMD) with the characteristic of interest. The direction and significance of these variables was unchanged when CMDs were excluded in the variable generation.
[e]Predictors were represented as the standard deviation of the characteristic of interest amongst the CMDs and their friends. The direction and significance of these variables was unchanged when CMDs were excluded in the variable generation.

Supplementary Table 10 Robustness of treatment speed to temporal clustering, CMD friendship, & homophily

	Univariate regressions[a]	*Dependent variable: Number of days to reach 50% household coverage[b]*				
	**Predictors**	**Coeff.**	**Robust Std. Err.**	**p-value**	**95% CI**	
**A) Temporal effects & CMD[c] friendship**	Std. Dev. of day of drug receipt for households that are friends with CMDs (Temporal clustering, excluding CMDs)	0.121	0.079	0.127	-0.034	0.276
	CMDs are directly connected in friendship network	0.020	0.228	0.931	-0.428	0.467
**B) Manifest homophily**	Shortest path (geodesic distance) between CMDs in friendship network	0.195	0.163	0.230	-0.124	0.515
	Average years as CMD is less than average years of friendship amongst friends of CMDs	0.141	0.276	0.610	-0.401	0.683
	Average years of friendship amongst friends of CMDs is less than MDA existence (10 years)	0.317	0.268	0.236	-0.208	0.842
	Average years as CMD	-0.010	0.022	0.650	-0.053	0.033
**C) Secondary homophily**	Household seeks medical care from private clinics[d]	-0.273	0.552	0.620	-1.355	0.808
	Social status: at least one household member was or is a religious, tribe, or clan leader or on the local village council[d]	-3.245	1.895	0.087	-6.958	0.468
	Muslim household head[d]	-0.342	1.163	0.769	-2.621	1.937
	Household head belongs to majority tribe[d]	-0.390	0.706	0.580	-1.773	0.993
	Household purifies drinking water[d]	0.115	0.348	0.741	-0.567	0.797
	Home latrine[d]	-1.181	0.992	0.234	-3.125	0.763
	Highest level of education attained by a household member[e]	-0.273	0.297	0.357	-0.855	0.308
	Total years household settled in village[e]	0.069	0.057	0.223	-0.042	0.180
	Home quality score[e]	-0.232	0.264	0.379	-0.748	0.285
	Household had electricity[d]	-0.892	1.221	0.465	-3.286	1.501
	Obs. 15[f]					

[a] Each model is a single predictor regression. Poisson regressions were used; constants are not shown.
[b] Household coverage is defined as the proportion of households in the village where at least one eligible person in the home was offered at least one treatment through mass drug administration.
[c] Community medicine distributor.
[d] Predictors were represented as the percentage of CMDs friends (including the CMD) with the characteristic of interest. The direction and significance of these variables was unchanged when CMDs were excluded in the variable generation.
[e] Predictors were represented as the standard deviation of the characteristic of interest amongst the CMDs and their friends. The direction and significance of these variables was unchanged when CMDs were excluded in the variable generation.
[f] Only 15/17 villages achieved 50% household coverage during the one-month distribution observed for this study.

Supplementary Table 10 presents possible confounders to the effects of CMD network properties on the speed of treatment. The table is a complement to Supplementary Table 9 (discussed in the main text), which presents the dependent variable of village treatment coverage. The results in Supplementary Table 10 are qualitatively consistent with Supplementary Table 9; the significance of all variables remained unchanged. Temporal clustering, CMD friendship, and homophily were insignificant (p-value>0.05) for the speed of treatment diffusion.

Supplementary Table 11 Robustness of treatment coverage and speed to CMD personal characteristics

**Univariate regressions**[a]	*A) Dependent variable: Household coverage*[b]					*B) Dependent variable: Number of days to reach 50% household coverage*[c]				
**Personal characteristics of CMDs**[d]	**Coeff.**	**Robust Std. Err.**	**p-value**	**95% CI**		**Coeff.**	**Robust Std. Err.**	**p-value**	**95% CI**	
Household seeks medical care from private clinics [e]	-0.268	0.234	0.252	-0.727	0.190	0.159	0.153	0.301	-0.142	0.459
Social status: at least one household member was or is a religious, tribe, or clan leader or on the local village council [e]	0.322	0.233	0.168	-0.135	0.779	-0.160	0.245	0.515	-0.641	0.321
Muslim household head [e]	-0.070	0.257	0.784	-0.573	0.432	0.126	0.188	0.503	-0.242	0.493
Household head who belongs to majority tribe [e]	-0.082	0.320	0.798	-0.708	0.545	0.689	0.425	0.105	-0.143	1.521
Household purifies drinking water [e]	0.264	0.245	0.282	-0.217	0.745	-0.010	0.177	0.955	-0.356	0.336
No home latrine[f]	-	-	-	-	-	-	-	-	-	-
Average of highest level of education attained by a household member[g]	0.054	0.037	0.145	-0.018	0.125	-0.055	0.032	0.089	-0.119	0.008
Average of total years household settled in village[g]	-0.019	0.020	0.325	-0.058	0.019	0.004	0.015	0.799	-0.025	0.032
Average home quality score[g]	-0.053	0.056	0.345	-0.163	0.057	-0.038	0.039	0.332	-0.115	0.039
Household electricity [e]	-0.490	0.314	0.118	-1.106	0.125	-0.162	0.174	0.351	-0.503	0.179
Obs. 17						Obs. 15[h]				

[a] Each model is a single predictor regression. Fractional response models were used with probit links for the dependent variable of household coverage; constants are not shown. Poisson regressions were used for the dependent variable of number of days to 50% household coverage; constants not shown.
[b] Household coverage is defined as the proportion of households in the village where at least one eligible person in the home was offered at least one treatment through mass drug administration.
[c] The number of days to reach 50% household coverage was recorded within a one-month distribution period for each village.
[d] CMD is an abbreviation for community medicine distributor.
[e] These binary variables were constructed as follows. The original variables for each CMD were equal to one if the CMD had the characteristic described. To assess the similarity in these characteristics between CMDs since the average of the binary indicators is not informative, the variables were coded as one if the CMDs both had different values for the variable. For example, for 'household purifies water', if one CMD's household indicated 'yes' and another CMDs' household indicated 'no' to purifying water then the binary indicator here would be equal to one. Hence, the base category for all the binary indicators is that there are no differences in the values of these variables between the two CMDs in each village.
[f] Ownership of a home latrine was not included as a predictor in the univariate regressions because there was no variation in this binary indicator. All CMDs had a home latrine.
[g] The variable was averaged for the two CMDs in each village.
[h] Only 15/17 villages achieved 50% household coverage during the one-month distribution observed for this study.

Supplementary Table 11 presents how the similarity between CMDs affects treatment diffusion. The table is a complement to Supplementary Table 9 (discussed in-text) and Supplementary Table 10. No observable characteristics of CMDs were associated (p-value>0.05) with the reach and speed of treatment diffusion.

Supplementary Table 12 Association of CMD clustering with CMD personal characteristics

	Dependent variable				
**Univariate regressions**[a]	**Clustering coefficient of CMD**[b]				
**Predictors**	**Coeff.**	**Robust Std. Err.**	**p-value**	**95% CI**	
Age	0.001	0.003	0.826	-0.006	0.008
Female	0.055	0.145	0.706	-0.230	0.339
Household seeks medical care from private clinics	0.263	0.138	0.056	-0.007	0.533
Social status: at least one household member was or is a religious, tribe, or clan leader or on the local village council	0.086	0.142	0.542	-0.191	0.364
Muslim household head	-0.054	0.141	0.700	-0.331	0.222
Household head belongs to majority tribe	-0.006	0.153	0.971	-0.306	0.295
Household purifies drinking water	0.088	0.140	0.531	-0.186	0.362
No home latrine[c]	-	-	-	-	-
Highest level of education attained by a household member[d]	<0.001	<0.001	0.964	<-0.001	<0.001
Total years household settled in village	-0.010	0.006	0.107	-0.023	0.002
Home quality score	0.014	0.016	0.403	-0.018	0.045

Obs. 34

[a]Fractional response models with probit links; constants not shown

[b]Community medicine distributor

[c]Omitted; no variation in this variable for CMDs. All CMDs had a home latrine.

[d]Adjusted for heteroskedasticity

Supplementary Table 13 Robustness of coverage amongst friends of CMDs to homophily

	Univariate regressions[a]	*Dependent variable: Household coverage amongst friends of CMDs*[b]				
	**Predictors**	**Coeff.**	**Robust Std. Err.**	**p-value**	**95% CI**	
**A) Manifest homophily**	Average years as CMDs was less than average years of friendship amongst friends of CMDs	-0.278	0.215	0.197	-0.700	0.144
	Average years of friendship amongst friends of CMDs was less than MDA existence (10 years)	0.079	0.367	0.829	-0.640	0.798
	Average years as CMD	0.024	0.055	0.661	-0.083	0.131
**B) Secondary homophily**	Household seeks medical care from private clinics[c]	0.934	0.584	0.110	-0.211	2.080
	Social status: at least one household member was or is a religious, tribe, or clan leader or on the local village council[c]	2.201	1.626	0.176	-0.986	5.388
	Muslim household head[c]	-1.608	1.137	0.157	-3.837	0.620
	Household head belongs to majority tribe[c]	-1.716	1.772	0.333	-5.190	1.757
	Household purifies drinking water[c]	-0.713	0.773	0.356	-2.228	0.802
	Home latrine[c]	0.476	1.295	0.713	-2.062	3.015
	Highest level of education attained by a household member[d]	0.048	0.215	0.822	-0.373	0.469
	Total years household settled in village[d]	-0.097	0.076	0.204	-0.246	0.053
	Home quality score[d]	-0.068	0.251	0.786	-0.560	0.424
	Household had electricity[c]	-2.696	2.635	0.306	-7.862	2.469
	Obs. 17					

[a] Each model is a single predictor regression. Fractional response models were used with probit links; constants are not shown.
[b] Household coverage is defined as the proportion of households in the village where at least one eligible person in the home was offered at least one treatment through mass drug administration. CMD stands for community medicine distributor.
[c] Predictors were represented as the percentage of CMDs friends (including the CMD) with the characteristic of interest. The direction and significance of these variables was unchanged when CMDs were excluded in the variable generation.
[d] Predictors were represented as the standard deviation of the characteristic of interest amongst the CMDs and their friends. The direction and significance of these variables was unchanged when CMDs were excluded in the variable generation.

Supplementary Table 13 shows that no relationships with manifest or secondary homophily for friends of CMDs were found (p-value>0.05) with the household coverage amongst those households.

Supplementary Table 14 Treatment outcomes against village size, accessibility, and ecology

Univariate regressions	*A) Dependent variable: household coverage*[a]					*B) Dependent variable: Number of days to reach 50% household coverage*[b]				
**Predictors**	**Coeff.**	**Robust Std. Err.**	**p-value**	**95% CI**		**Coeff.**	**Robust Std. Err.**	**p-value**	**95% CI**	
Total households in village	-0.002	0.001	0.077	-0.004	<0.001	0.002	0.001	0.054	<-0.001	0.004
Fraction of total households connected to CMDs[c]	0.214	0.707	0.762	-1.172	1.600	-1.177	0.752	0.117	-2.650	0.296
Maximum distance (meters) btw households[d]	<-0.001	<0.001	0.815	0.000	<0.001	<0.001	<0.001	0.810	<-0.001	0.001
Top 10th percentile of distance (meters) btw households[d]	<-0.001	<0.001	0.901	-0.001	<0.001	<-0.001	<0.001	0.837	-0.001	0.001
Avg. distance (meters) btw households[d]	<-0.001	0.001	0.966	-0.001	0.001	<0.001	0.001	0.995	-0.002	0.002
Rice paddy (large rice farm) within village	0.326	0.356	0.360	-0.372	1.024	0.005	0.120	0.967	-0.231	0.241
Beach on Lake Victoria within village	-0.109	0.242	0.652	-0.584	0.366	-0.007	0.216	0.975	-0.429	0.416
Lake site only within village (no beach, but small boat landing site) on Lake Victoria	-0.179	0.297	0.548	-0.761	0.404	0.112	0.183	0.542	-0.247	0.470
Village centre more than 0.50 km to Lake Victoria	-0.252	0.218	0.249	-0.680	0.177	0.115	0.196	0.558	-0.269	0.499
3 or more roads within village	0.105	0.251	0.677	-0.388	0.597	0.237	0.184	0.198	-0.124	0.598
CMDs also distribute treatment from their home[e]	0.167	0.253	0.510	-0.330	0.663	-0.410	0.235	0.081	-0.871	0.051
	Obs. 17					Obs. 15[f]				

[a] Each model is a single predictor regression. Fractional response models were used with probit links; constants are not shown. Household coverage is defined as the proportion of households in the village where at least one eligible person in the home was offered at least one treatment through mass drug administration.
[b] Each model is a single predictor regression. Poisson regressions were used; constants are not shown. The number of days to reach 50% household coverage was recorded within a one-month distribution period for each village.
[c] CMD stands for community medicine distributor.
[d] These village-level variables were calculated from the distance in meters between every two households in a village.
[e] CMDs were asked if they used any form of distribution, in addition to the home-to-home distribution that was the instructed method during national training. One other form of distribution was stated; CMDs allowed individuals to pick-up treatment from the CMDs' homes. This variable was equal to one if CMDs allowed pick-up of treatment.
[f] Only 15/17 villages achieved 50% household coverage within the one-month distribution period of this study.

Supplementary Table 14 presents the contribution of village size, accessibility, and ecology to treatment coverage and treatment speed achieved by CMDs. These results are discussed in the main text. The total households in the village, which included 55 interviewed households outside of the friendship network and 87 households that refused to be interviewed, was insignificant (p-value>0.05) for both household and individual coverage. The fraction of total households in the village that were friends with CMDs also was insignificant (p-value>0.05) for treatment coverage. Other variables that measured the distance between households in a village (accessibility of the village for CMDs), village ecology, and other forms of treatment distribution were all uncorrelated (p-value>0.05) with treatment coverage and speed.

Supplementary Table 15 Households without day of drug receipt information by village

Village ID	Eligible households in village	Eligible households amongst CMD friends [a]	Non-complying households in village	*Proportion non-complying households*	Non-complying households amongst CMD friends [a]	*Proportion of non-complying households amongst eligible CMD friends* [a]	Households in village receiving treatment but do not know day of drug receipt	*Proportion households where no one remembered day of drug receipt*	Households receiving treatment but do not know day of drug receipt amongst CMD friends [a]	*Proportion households where no one remembered day of drug receipt amongst eligible CMD friends* [a]
1	200	23	19	0.095	1	0.043	2	0.010	0	0
2	180	41	2	0.011	0	0	0	0	0	0
3	190	55	32	0.168	6	0.109	2	0.011	0	0
4	318	44	16	0.050	4	0.091	11	0.035	1	0.023
5	183	49	5	0.027	2	0.041	5	0.027	1	0.020
6	139	61	2	0.014	0	0	0	0	0	0
7	121	46	8	0.066	2	0.043	0	0	0	0
8	367	48	38	0.104	5	0.104	17	0.046	2	0.042
9	178	107	1	0.006	0	0	2	0.011	0	0
10	204	77	24	0.118	5	0.065	4	0.020	2	0.026
11	244	30	10	0.041	2	0.067	0	0	0	0
12	229	72	14	0.061	3	0.042	8	0.035	3	0.042
13	183	44	7	0.038	2	0.045	9	0.049	0	0
14	122	37	2	0.016	0	0	1	0.008	0	0
15	120	26	7	0.058	0	0	3	0.025	1	0.038
16	372	61	31	0.083	4	0.066	18	0.048	2	0.033
17	65	22	10	0.154	5	0.227	3	0.046	0	0
**Total**	3415	843	228	0.067	41	0.049	85	0.025	12	0.014

[a] CMD is an abbreviation for community medicine distributor. These variables also include the CMDs households.

Supplementary Table 15 presents the breakdown of households where the day of drug receipt was unknown, although treatment was offered by CMDs.

Supplementary Table 16 Balanced wealth/poverty across study villages

Selection factors	Avg. highest level of education attained by a household member	*Std. Dev.*	Avg. total years household settled in village	*Std. Dev.*	Avg. home quality score	*Std. Dev.*	Proportion of households with electricity	*Freq.*
**1**	6.515	*2.459*	16.005	*13.602*	6.153	*3.104*	0.0050	*1/202*
**2**	6.746	*2.733*	11.674	*9.244*	6.569	*2.848*	0.0442	*8/181*
**3**	6.656	*3.218*	15.422	*11.841*	6.594	*3.807*	0.0625	*12/192*
**4**	6.206	*2.903*	13.613	*10.859*	4.888	*2.781*	0.1281	*41/320*
**5**	6.614	*2.465*	13.962	*12.409*	6.060	*3.532*	0.0924	*17/184*
**6**	6.547	*2.839*	18.770	*12.283*	7.568	*2.375*	0.0360	*5/139*
**7**	6.719	*2.757*	16.000	*12.066*	6.620	*2.922*	0.061	*8/121*
**8**	6.938	*2.943*	12.314	*9.630*	6.564	*3.815*	0.0163	*6/369*
**9**	7.034	*3.329*	12.000	*9.466*	6.775	*3.807*	0.0787	*14/178*
**10**	6.689	*2.908*	16.325	*11.209*	5.797	*3.268*	0.0338	*7/207*
**11**	6.600	*2.858*	12.736	*11.354*	6.932	*3.158*	0.0520	*13/250*
**12**	8.140	*3.190*	14.017	*11.789*	8.035	*2.730*	0.1179	*27/229*
**13**	7.306	*3.334*	16.355	*13.308*	7.749	*3.681*	0.0383	*7/183*
**14**	7.089	*2.538*	11.460	*9.280*	6.185	*3.184*	0.0565	*7/124*
**15**	7.033	*2.432*	15.483	*11.203*	6.483	*3.207*	0.033	*4/120*
**16**	7.752	*2.923*	10.487	*7.958*	6.774	*3.685*	0.0618	*23/372*
**17**	6.359	*3.297*	10.600	*8.725*	5.015	*3.664*	0.0462	*3/65*

Supplementary Table 16 presents the balance of infrastructural development across the study villages, which was captured in the variables of home quality (also indicative of wealth), availability of electricity, education, and the longevity of the village (years of residence for villagers). The number of observations for the variables where the average was presented was the same as the observations presented in the denominator of the frequency column. The study villages display similar properties across these characteristics.

Supplementary Table 17 Association of CMD clustering coefficients with homophily

	Univariate regressions[a]	*Dependent variable: Average clustering coeff. of CMDs[b]*				
	**Predictors**	**Coeff.**	**Robust Std. Err.**	**p-value**	**95% CI**	
**A) Manifest homophily**	Average years as CMD is less than average years of friendship amongst friends of CMDs	-0.225	0.223	0.313	-0.663	0.212
	Average years of friendship amongst friends of CMDs is less than MDA existence (10 years)	-0.265	0.194	0.171	-0.644	0.114
	Average years as CMD	-0.017	0.025	0.498	-0.065	0.032
**B) Secondary homophily**	Household seeks medical care from private clinics[c,e]	0.789	0.188	<0.001	0.422	1.157
	Social status: at least one household member was or is a religious, tribe, or clan leader or on the local village council[c]	3.546	0.817	<0.001	1.944	5.148
	Muslim household head[c]	0.277	0.584	0.635	-0.868	1.423
	Household head belongs to majority tribe[c]	0.017	0.627	0.978	-1.211	1.246
	Household purifies drinking water[c]	-0.110	0.230	0.633	-0.560	0.341
	Home latrine[c]	2.254	1.021	0.027	0.253	4.256
	Highest level of education attained by a household member[d,e]	0.212	0.059	<0.001	0.095	0.328
	Total years household settled in village[d]	-0.049	0.052	0.347	-0.150	0.053
	Home quality score[d]	0.201	0.251	0.422	-0.290	0.693
	Household had electricity[c]	2.171	1.459	0.137	-0.689	5.031
	Obs. 17					

[a] Each model is a single predictor regression. Fractional response models were used with probit links; constants are not shown.
[b] CMD stands for community medicine distributor.
[c] Predictors were represented as the percentage of CMDs friends (including the CMD) with the characteristic of interest. The direction and significance of these variables was unchanged when CMDs were excluded in the variable generation.
[d] Predictors were represented as the standard deviation of the characteristic of interest amongst the CMDs and their friends. The direction and significance of these variables was unchanged when CMDs were excluded in the variable generation.
[d] Adjusted for heteroskedasticity.

Supplementary Table 17 presents the association of homophily with the clustering coefficient of CMDs. The average clustering coefficient of CMDs was used as the dependent variable to enable comparisons with the main text where this variable was a predictor for diffusion. Although there is no theoretical basis for why mass drug administration may affect the formation of friendship ties, these variables were examined as a robustness check. No variables representing manifest homophily were associated with the average clustering coefficient of CMDs. However, it is well-established that clustering within social networks is due largely due to homophily [22], i.e. tightly-knit groups form that share similar characteristics. Our networks confirm this knowledge. Amongst CMDs and their friends, increased likeness on social status (p-value<0.001), education (p-value<0.001), latrine ownership (p-value=0.027), and use of private medical care (p-value<0.001) was positively associated with clustering around CMDs. These results further confirm the internal validity of our data.

Supplementary Table 18 Local transitivity robustness against multiple hypotheses

Univariate regressions	*A) Dependent variable: household coverage* [a]					*B) Dependent variable: Number of days to reach 50% household coverage* [b]				
**Predictors**	**Coeff.**	**Robust Std. Err.**	**p-value**	**95% CI**		**Coeff.**	**Robust Std. Err.**	**p-value**	**95% CI**	
Local transitivity factor	0.216	0.105	0.040	0.010	0.422	-0.410	0.147	0.005	-0.698	-0.122
Centrality factor	-0.034	0.110	0.755	-0.249	0.180	-0.087	0.084	0.300	-0.252	0.078
Centrality factor without degree	-0.019	0.107	0.859	-0.229	0.191	-0.094	0.083	0.253	-0.256	0.067
Naïve local transitivity factor	4.751	2.295	0.038	0.252	9.250	-8.426	3.067	0.006	-14.437	-2.415
Naïve centrality factor	-0.047	0.050	0.349	-0.146	0.052	-0.002	0.045	0.965	-0.090	0.087
Naïve centrality factor without degree	-0.115	1.252	0.927	-2.570	2.339	-1.276	0.978	0.192	-3.193	0.642
	Obs. 17					Obs. 15 [c]				

[a] Each model is a single predictor regression. Fractional response models were used with probit links; constants are not shown. Household coverage is defined as the proportion of households in the village where at least one eligible person in the home was offered at least one treatment through mass drug administration.
[b] Each model is a single predictor regression. Poisson regressions were used; constants are not shown. The number of days to reach 50% household coverage was recorded within a one-month distribution period for each village.
[c] Only 15/17 villages achieved 50% household coverage within the one-month distribution period of this study.

As a robustness check against multiple hypotheses, we perform a confirmatory principal component analysis [36] on our average CMD network indicators then rerun our regressions on diffusion reach and speed. This analysis was done to reduce the data into two dimensions: local transitivity and centrality. Two indices were constructed to measure a set of conceptually similar variables. Instead of using each indicator of transitivity (average CMD: clustering, ego-network density, and ego-network reciprocity) as a predictor of diffusion, we construct a factor variable relating all of these measures. Another factor is constructed for the measures of centrality (average CMD: degree, avg. neighbor degree, closeness, eigenvector, katz, betweenness, and communicability). All local transitivity factors are uncorrelated with the centrality factors (p-value>0.05). Thus, we have now collapsed our predictors into a smaller set of variables (two predictors/factors) that are uncorrelated with each other rather than using a large set of variables that are correlated (i.e. the correlation between different centrality measures, though clustering and centrality are not correlated Supplementary Table 6). We now use these factors as predictors of diffusion. Additionally, we construct a second version of the centrality factor without degree and average neighbor degree. Lastly, to further demonstrate the validity of our hypotheses, we also construct naïve indices of local transitivity and centrality. The naïve index for local transitivity aggregates average CMD clustering, ego-network density, and ego-network reciprocity and divides by the number of variables (here, three). Naïve indices were constructed for centrality, with and without degree and average neighbor degree.

Most importantly, all results remained qualitatively the same. Both indices/factors of local transitivity retained the signs of the coefficient found in the main paper and remained significant (p-value<0.05) against diffusion reach and speed. Both indices/factors of centrality remained insignificant (p-value>0.05) against household coverage and speed of treatment.

Supplementary Table 19 Reach of household coverage adjusted for missing information on day of treatment offer

Univariate regressions[a]	*Dependent variable: Household coverage*				
**Average CMD[b] network properties**	**Coeff.**	**Robust Std. Err.**	**p-value**	**95% CI**	
Clustering	2.038	0.950	0.032	0.175	3.900
Reciprocity of CMD ego-network[c]	61.948	38.785	0.110	-14.070	137.965
Density of CMD ego-network	4.147	2.457	0.091	-0.669	8.963
Degree	-0.008	0.010	0.408	-0.027	0.011
Avg. neighbour degree	-0.024	0.032	0.453	-0.086	0.038
Core number	-0.028	0.060	0.644	-0.146	0.090
Clique number	0.014	0.117	0.908	-0.217	0.244
Closeness	0.598	1.503	0.691	-2.348	3.543
Eigenvector	0.048	1.802	0.979	-3.483	3.579
Katz	0.493	1.346	0.714	-2.145	3.132
Betweenness	-2.663	4.485	0.553	-11.454	6.127
Communicability	-0.117	0.642	0.856	-1.375	1.141

Obs. 17

[a]Fractional response models with probit link; constants not shown. The dependent variable is the proportion of households where at least one eligible individual was offered at least one treatment.

[b]Community medicine distributor

[c]Adjusted for heteroskedasticity

As a robustness check, we repeated the analysis of the reach of treatment coverage with household coverage adjusted for missing data. We removed the 228 households for which we did not have information on the day of drug receipt from the numerator and denominator when calculating household coverage. This table is presented to enable comparisons with the analysis of treatment speed, in particular because households that did not have information on the day of drug offer were missing from the analysis of treatment speed. All results presented in the main text remained.

Supplementary Table 20 Coefficient stability analysis

Covariate added[a]	Controlled effect of clustering	Robust std. err.	p-value	R-squared	Identified set		δ for clustering effect=0
Degree	0.589	0.311	0.079	0.095	0.553	0.589	6.607
Avg. neighbour degree	0.757	0.341	0.044	0.089	0.757	0.862	-1.120
Core number	0.701	0.306	0.038	0.073	0.701	0.800	-0.964
Clique number	0.724	0.378	0.076	0.064	0.724	1.035	0.223
Closeness	0.719	0.451	0.133	0.062	0.719	1.176	0.373
Eigenvector	0.643	0.310	0.057	0.059	0.643	0.788	-0.252
Katz	0.623	0.287	0.047	0.060	0.494	0.623	1.933
Betweenness	0.627	0.281	0.042	0.102	0.621	0.627	37.814
Communicability	0.682	0.348	0.070	0.067	0.682	0.786	-0.796
Household seeks medical care from private clinics [b]	0.542	0.291	0.084	0.102	0.477	0.542	4.123
Social status: at least one household member was or is a religious, tribe, or clan leader or on the local village council [b]	0.533	0.363	0.164	0.115	0.471	0.533	4.499
Muslim household head [b]	0.628	0.298	0.054	0.058	-0.313	0.628	0.918
Household head who belongs to majority tribe [b]	0.727	0.528	0.190	0.062	0.727	1.189	0.370
Household purifies drinking water [b]	0.563	0.353	0.133	0.112	0.518	0.563	5.901
Average of highest level of education attained by a household member	0.490	0.348	0.180	0.071	0.243	0.490	1.424
Average of total years household settled in village	0.609	0.283	0.049	0.098	0.588	0.609	11.362
Average home quality score	0.965	0.464	0.056	0.180	0.965	1.111	-1.383
Household electricity [b]	0.688	0.361	0.078	0.254	0.688	0.708	-17.384

Obs. 17

[a] Average Community medicine distributor (CMD) characteristic unless otherwise noted.

[b] These binary variables were constructed as follows. The original variables for each CMD were equal to one if the CMD had the characteristic described. To assess the similarity in these characteristics between CMDs since the average of the binary indicators is not informative, the variables were coded as one if the CMDs both had different values for the variable. For example, for 'household purifies water', if one CMD's household indicated 'yes' and another CMDs' household indicated 'no' to purifying water then the binary indicator here would be equal to one. Hence, the base category for all the binary indicators is that there are no differences in the values of these variables between the two CMDs in each village.

No evidence was found to indicate that unobservable factors (omitted variable biases) were driving the association of average CMD clustering with household coverage. We implement methods described in Oster[37] and Antonji *et al*[38] using Stata v13.1 with the package psacalc[37]. These approaches examine the coefficient stability of the predictor of interest, here clustering, when additional covariates are added and the subsequent movements in R^2. Such analysis is necessary if the insignificance of covariates does not capture the effect of unobservables. However, assessments of coefficient stability to infer omitted variable bias ultimately rely on a strong assumption that observables and unobservables similarly affect outcomes. Another important assumption is the maximum R^2, $R_{max}[0,1]$, that can be achieved in a controlled (with covariates) model. We run ordinary least squares regressions with the dependent variable of household coverage and covariates of centrality and personal characteristics of CMDs; factors we argue are irrelevant for treatment coverage. Home latrine was excluded, as there was no variation in this variable for CMDs; all CMDs had a home latrine. No added covariates were significant (here, p-value<0.10) in any of the regressions; this result accords with the univariate analysis. We chose R_{max} as $1.3*R^2_{controlled}$ where controlled represented R^2 from the controlled regression, as recommended in Oster[37]. However, all results were qualitatively the same when $2.2*R^2_{controlled}$ was used. Each controlled regression included a single covariate in addition to average CMD clustering. We limit each model to 2 predictors due to the limited number of observations (17). In the uncontrolled model, with only the predictor of average CMD clustering, this variable had a coef.= 0.637,, std. err.= 0.270, R^2= 0.058, and p-value=0.032. We provide the estimated range for the coefficient of clustering after any adjustment for unobservable influences (identified set). This identified set always excluded 0 (except in the case of CMD religion where $R^2_{controlled}$ was exactly unchanged from the controlled model (0.058)), indicating a positive influence of clustering.

With the addition of another covariate and, in turn, noise to the model, statistical power was lost and p-values expectedly were not retained always below 0.05. We also provide the estimate of δ required for the coefficient of clustering to equal 0 or to be 'eliminated.' Antonji *et al.*[38] suggest a cutoff of δ=1 as robust; anything above this cutoff indicates that the unobservables must have a stronger effect than the observable factors to exert bias. A negative δ suggests that the addition of covariates actually strengthens the coefficient of clustering as opposed to attenuating it towards 0. In 4 cases, for CMD religion, tribe, clique number, and closeness, was δ positive and below 1. However, in these cases, $R^2_{controlled}$ either was completely unchanged (religion) or virtually unchanged from the R^2 of the uncontrolled model, a setting where the Oster[37] approach is not applicable and indicative of likely capturing noise.

Supplementary Table 21 Coefficient stability analysis continued

Two covariates added	Controlled effect of clustering	Robust std. err.	p-value	R-squared	Identified set		δ for clustering effect=0
Closeness; Average of highest level of education attained by a household member	0.581	0.502	0.267	0.075	0.354	0.581	1.219
Degree; Household purifies drinking water	0.525	0.403	0.216	0.142	0.469	0.525	5.383
Household head who belongs to majority tribe; Average of highest level of education attained by a household member	0.565	0.561	0.333	0.072	0.062	0.565	1.027
Clique number; Average of total years household settled in village	0.748	0.430	0.106	0.111	0.748	0.839	15.936
Closeness; Average neighbor degree	0.705	0.491	0.175	0.091	0.705	0.798	2.544
Clique number; Household seeks medical care from private clinics	0.636	0.356	0.097	0.108	0.636	0.636	3.298
Muslim household head; Average of total years household settled in village	0.606	0.325	0.085	0.098	0.582	0.606	5.322
Eigenvector; Muslim household head	0.746	0.565	0.210	0.063	0.746	1.166	0.365
Clique number; Average neighbor degree	0.717	0.441	0.128	0.091	0.717	0.808	4.739
Household seeks medical care from private clinics; Average of highest level of education attained by a household member	0.424	0.379	0.284	0.111	0.263	0.424	1.885

All network variables are averages for CMDs. For socioeconomic variables, if not indicated as an average, then these variables were binary. The binary variables were constructed as follows. The original variables for each CMD were equal to one if the CMD had the characteristic described. To assess the similarity in these characteristics between CMDs since the average of the binary indicators is not informative, the variables were coded as one if the CMDs both had different values for the variable. For example, for 'household purifies water', if one CMD's household indicated 'yes' and another CMDs' household indicated 'no' to purifying water then the binary indicator here would be equal to one. Hence, the base category for all the binary indicators is that there are no differences in the values of these variables between the two CMDs in each village.

The approach employed in Supplementary Table 20 was repeated here with a uniform random selection (in R v3.3.3) of two covariates instead of one covariate to run alongside clustering though underpowered. There are 120 combinations of two covariates from 16 predictors, so we present a random sample of 10 from the 120 regressions. Other combinations did not change the conclusions of this study. Clustering remained positive and greater than zero.

Supplementary References

1. Kabatereine, N. B., Brooker, S., Tukahebwa, E. M., Kazibwe, F. , Onapa, A. W., Epidemiology and geography of Schistosoma mansoni in Uganda: implications for planning control. *Trop. Med. Int. Health* **9**, 372-380 (2004).
2. Kabatereine, N.B., *et al*., Short communication: Soil-transmitted helminthiasis in Uganda: epidemiology and cost of control. *Trop. Med. Int. Health* **10**, 1187-1189 (2005).
3. " Preventive Chemotherapy in Human Helminthiases, Coordinated Use of Anthelminthic Drugs in Control Interventions: A manual for Health Professionals and Programme Managers," (WHO, Geneva, CH, 2006).
4. Forman, R., Godron, M., *Landscape ecology*. (New York, John Wiley & Sons, New York City, NY, 1986).
5. Schult, D. A., Swart, P., paper presented in Proceedings of the 7th Python in Science Conferences. Pasenda, CA, 19-24 August 2008
6. Newman, M., *Networks: an introduction*. (Oxford University Press, Oxford, UK, 2010).
7. Bonacich, P., Power and centrality: A family of measures. *Am. J. Sociol.* **92**, 1170-1182 (1987).
8. Katz, L., A new status index derived from sociometric analysis. *Psychometrika* **18**, 39-43 (1953).
9. Freeman, L. C. Centrality in social networks conceptual clarification. *Soc. Networks.* **1**, 215-239 (1979).
10. Estrada, E., Higham, D. J., Hatano, N., Communicability betweenness in complex networks. *Phys. A* **388**, 764-774 (2009).
11. Brandes, U., A faster algorithm for betweenness centrality. *J. Math. Sociol.* **25**, 163-177 (2001).
12. Saramaki, J., Kivela, M., Onnela, J.-P., Kaski, K., Kertesz, J., Generalizations of the clustering coefficient to weighted complex networks. *Phys. Rev. E* **75**, 027105 (2007).
13. Beaman, L., BenYishay, A., Magruder, J. & Mobarak, A. M. Can network theory based targeting increase technology adoption. Unpublished Manuscript (2016).
14. Batagelj, V., Zaversnik, M., An O (m) algorithm for cores decomposition of networks. *ADAC* **5**, 129-145 (2011).
15. Bron, C., Kerbosch, J., Algorithm 457: finding all cliques of an undirected graph. *Commun. ACM* **16**, 575-577 (1973).
16. Chami, G. F., Molyneux, D. H., Kontoleon, A. A., Dunne, D. W. , Exploring network theory for mass drug administration. *Trends Parasitol*. **29**, 370-379 (2013).
17. Papke, L. E., Wooldridge, J. M., Econometric methods for fractional response variables with an application to 401(k) plan participation rates. *J. Applied Econom.* **11**, 619-632 (1996).
18. Williams, R. L., A note on robust variance estimation for cluster, correlated data. *Biometrics* **56**, 645-646 (2000).
19. Cameron, A. C., Trivedi, P. K., *Microeconometrics using stata* (vol. 5, Stata Press College Station, TX, 2010) [revised edition].
20. Laird, N., Olivier, D., Covariance analysis of censored survival data using log-linear analysis techniques. *J. Am. Statist. Associ.* **76**, 231-240 (1981).
21. "Schistosomiasis and soil-transmitted helminth infections," Fifty-Fourth World Health Assembly (WHO, Geneva, CH, 2001).
22. McPherson, M., Smith-Lovin, L. & Cook, J. M. Birds of a feather: homophily in social networks. *Annu. Rev. Sociol*. **27**, 415-444 (2001).
23. Aral, S., Muchnik, L. & Sundararajan, A. Distinguishing influence-based contagion from homophily-driven diffusion in dynamic networks. *Proc. Natl. Acad. Sci.* U.S.A. **106**, 21544-21549, (2009).
24. Shalizi, C. R., Thomas, A. C., Homophily and contagion are generically confounded in observational social network studies. *Socio. Meth. Res.* **40**, 211-239 (2011).
25. R Core Team. R: A language and environment for statistical computing. (R Foundation for Statistical Computing, Vienna, Austria, 2014).
26. Friedman, J., Hastie, T. & Tibshirani, R. Regularization Paths for Generalized Linear Models via Coordinate Descent. *J Stat Softw* **33**, 1-22 (2010).
27. Chernozhukov, V., Hansen, C. & Spindler, M. hdm: High-Dimensional Metrics. arXiv preprint arXiv:1608.00354 (2016).
28. Hoerl, A. E. & Kennard, R. W. Ridge regression: Biased estimation for nonorthogonal problems. *Technometrics* **12**, 55-67 (1970).

29. Tibshirani, R. Regression shrinkage and selection via the lasso. *J R Stat Soc Series B Stat Methodol* **58,** 267-288 (1996).
30. Zou, H. & Hastie, T. Regularization and variable selection via the elastic net. *J R Stat Soc Series B Stat Methodol* **67**, 301-320 (2005).
31. Belloni, A., Chen, D., Chernozhukov, V. & Hansen, C. Sparse models and methods for optimal instruments with an application to eminent domain. *Econometrica* **80**, 2369-2429 (2012).
32. Landsat Level 1 Dataset, U.S. Geological Survey. NASA EOSDIS Land Processes DAAC, USGS Earth Resources Observation and Science (EROS) Center, Sioux Falls, South Dakota (2017). http://lpdaac.usgs.gov. Accessed May 2015.
33. Albert, R., Barabási, A.-L., Statistical mechanics of complex networks. *Rev. Mod. Phys.* **74**, 50 (2002).
34. Alstott, J., Bullmore, E., Plenz, D., Powerlaw: a Python package for analysis of heavy-tailed distributions. *PLOS ONE* **9**, e85777 (2014).
35. D'Agostino, R. B., Belanger, A. & D'Agostino, R. B., Jr. A suggestion for using powerful and informative tests of normality. *Am. Stat.* **44**, 316-321, (1990).
36. Basilevsky, A. T., *Statistical factor analysis and related methods: theory and applications.* (John Wiley & Sons, New York, New York, 2009).
37. Oster, E. Unobservable Selection and Coefficients Stability: Theory and Evidence. *J Bus Econ Stat*, Forthcoming. (2016).
38. Altonji, J. G., Elder, T. E. & Taber, C. R. Selection on observed and unobserved variables: Assessing the effectiveness of Catholic schools. *J. Polit. Econ.* **113**, 151-184 (2005).
